# Plant-derived chimeric antibodies inhibit the invasion of human fibroblasts by *Toxoplasma gondii*

**DOI:** 10.7717/peerj.5780

**Published:** 2018-12-11

**Authors:** Sherene Swee Yin Lim, Kek Heng Chua, Greta Nölke, Holger Spiegel, Wai Leong Goh, Sek Chuen Chow, Boon Pin Kee, Rainer Fischer, Stefan Schillberg, Rofina Yasmin Othman

**Affiliations:** 1 Institute of Biological Sciences, University of Malaya, Kuala Lumpur, Malaysia; 2 Department of Biomedical Science, Faculty of Medicine, University of Malaya, Kuala Lumpur, Malaysia; 3 Fraunhofer Institute for Molecular Biology and Applied Ecology IME, Aachen, Germany; 4 School of Science, Monash University Malaysia, Bandar Sunway, Selangor, Malaysia; 5 Centre for Research in Biotechnology for Agriculture, University of Malaya, Kuala Lumpur, Malaysia

**Keywords:** Parasite, IgG, Plantibodies, Phage display, scFv, Tachyzoites

## Abstract

The parasite *Toxoplasma gondii* causes an opportunistic infection, that is, particularly severe in immunocompromised patients, infants, and neonates. Current antiparasitic drugs are teratogenic and cause hypersensitivity-based toxic side effects especially during prolonged treatment. Furthermore, the recent emergence of drug-resistant toxoplasmosis has reduced the therapeutic impact of such drugs. In an effort to develop recombinant antibodies as a therapeutic alternative, a panel of affinity-matured, *T. gondii* tachyzoite-specific single-chain variable fragment (scFv) antibodies was selected by phage display and bioinformatic analysis. Further affinity optimization was attempted by introducing point mutations at hotspots within light chain complementarity-determining region 2. This strategy yielded four mutated scFv sequences and a parental scFv that were used to produce five mouse–human chimeric IgGs in *Nicotiana benthamiana* plants, with yields of 33–72 mg/kg of plant tissue. Immunological analysis confirmed the specific binding of these plant-derived antibodies to *T. gondii* tachyzoites, and in vitro efficacy was demonstrated by their ability to inhibit the invasion of human fibroblasts and impair parasite infectivity. These novel recombinant antibodies could therefore be suitable for the development of plant-derived immunotherapeutic interventions against toxoplasmosis.

## Introduction

*Toxoplasma gondii* is an obligate intracellular protozoan parasite that can invade and colonize almost any nucleated cell, resulting in life-long chronic infections that currently affect more than 30% of the world’s population (Manger, Hehl & Boothroyd, 1998; Montoya & Liesenfeld, 2004). Toxoplasmosis infections in immunocompetent individuals are usually self-limiting and are quickly resolved by immune system, thus preventing acute infection. However, *T. gondii* can cause severe neurological birth defects when transmitted congenitally during a primary infection, with a devastating life-long impact on child health, development, and later productivity (Holliman, 1995). The parasite is also an opportunistic pathogen affecting immunocompromised individuals such as AIDS patients, by causing the reactivation of latent infection into fulminant disease, usually manifested as toxoplasmic encephalitis and chorioretinitis (Israelski et al., 1993; Luft et al., 1993; Holliman, 1995; Hunter & Sibley, 2012; McLeod et al., 2012).

The current gold-standard drugs for toxoplasmosis cause inflammatory side effects and are generally unaffordable in less developed nations (Mui et al., 2008). Such drugs are also teratogenic and therefore cannot be used during the first trimester of pregnancy (Goldstein, Montoya & Remington, 2008). These factors combined with the emergence of drug-resistant *T. gondii* strains (Sims, 2009) have created a pressing need for novel antiparasitic therapies. Passive immunization is a useful alternative strategy particularly for diseases characterized by chronic, life-long infections. Vaccination strategies have enjoyed little success because the parasites can evade the immune system, and the major challenge has been to establish sterile immunity, which is an elusive vaccination endpoint (Lim & Othman, 2014). Although preventive vaccines are desirable, most lethal cases of toxoplasmosis are reactivated infections in immunocompromised patients with insufficient cellular immunity (Luft & Remington, 1992). Recombinant antibodies are suitable in this context for passive immunization to inhibit pathogenesis, either as a replacement or adjunct for conventional interventions. Neutralizing antibodies against *T. gondii* have already demonstrated the feasibility of this strategy (Cha et al., 2001; Fu et al., 2011).

Plants have emerged as a competitive expression platform for recombinant antibodies because they reduce the cost of production (Ma & Hein, 1995; Fischer & Emans, 2000; Fischer et al., 2013; Twyman, Schillberg & Fischer, 2013; Kim et al., 2016). Plants also achieve the efficient assembly of complex multimeric antibodies closely resembling those produced in mammalian systems, but there is no risk of exposure to animal-derived products and pathogens. Recombinant antibodies derived from murine sources can induce an undesirable human anti-mouse antibody response. Strategies to overcome this issue include antibody humanization (Mateo et al., 1997), phage-display chain shuffling (Kupper et al., 2005), chimeric antibody construction or complementarity-determining region (CDR) grafting (Vaquero et al., 1999).

Previous studies had demonstrated successful production of anti-parasite antibodies in plants and their potential as new therapeutic treatments (Voepel et al., 2014; Chen et al., 2016; Fragoso et al., 2017). In this study, we aim to develop and convert a *T. gondii*-specific single-chain variable fragment (scFv) antibody from a murine source into a chimeric human IgG and produce a functional full-size molecule by expression in *Nicotiana benthamiana* and further showing the plant-derived chimeric antibodies can specifically bind to the parasite and reduce the infectivity.

## Materials and Methods

### Parasites and in vivo passaging in mice

All parasite and cell culture procedures were carried out in class II type A2 lamina flow hoods. The Hs68 fibroblast cell line derived from human foreskin fibroblast (HFF) was obtained from ATCC (CRL-1446). The cells were cultured in Dulbecco’s Modified Eagle’s Medium (DMEM) supplemented with 10% (v/v) fetal calf serum (FCS), two mM L-glutamine, 100 units/ml penicillin, and 100 μg/ml streptomycin. When the cells were 100% confluent, they were maintained in infection medium (as above, but with 1% (v/v) FCS).

The Hs68 cells were inoculated with 4 × 10^6^ *T. gondii* tachyzoite cells and incubated in a humidified cell culture chamber at 37 °C with a 5% CO_2_ atmosphere. After 48 h, most of the *T. gondii* tachyzoites were egressed from the host cells. A cell scrapper was used to disrupt cells containing rosettes (tachyzoites) and the tachyzoites were passed through a 27-gauge needle and centrifuged at 1,000×*g* for 15 min. The pellet containing tachyzoites was resuspended in five ml infection medium and filtered through a three-μm Nucleopore polycarbonate filter membrane (GE Healthcare Life Sciences, Marlborough, MA, USA) to remove cell debris. The filtered parasites were then quantified with a hemocytometer and trypan blue staining (Sambrook & Russell, 2001). Native soluble antigen (CAT. No: EV8131) from lysed *T. gondii* was acquired commercially (Meridian Life Science, Memphis, TN, USA).

### Mouse immunization

Three BALB/c mice, 6–8 weeks old, were immunized by the subcutaneous injection of 2.02–4.04 × 10^6^ *T. gondii* RH tachyzoites emulsified in Freund’s Complete Adjuvant (CAT. No: F5881) (Sigma-Aldrich, St. Louis, MO, USA) at a ratio of 1:1, with an interval of 2 weeks (day 0 and day 14), followed by a second booster injection on day 21 with the same amount of tachyzoites but this time without adjuvant. The immunized mice were bled 4 days after the second booster and the serum was tested for the presence of *T. gondii*-specific IgG antibodies by immunoblot analysis (Towbin, Staehelin & Gordon, 1979). All methodologies were carried out in accordance with University of Malaya, Animal Care and Use Ethical Committee (ACUC) guidelines and all experimental protocols were approved by the ACUC of the University of Malaya (Clearance number: PAR/18/06/2007/FMY(R). Following the detection of *T. gondii*-specific IgG antibodies, mouse spleens were harvested, snap-frozen in liquid nitrogen and stored at −80 °C.

### Construction of the phage–scFv display library

Total RNA was prepared from homogenized mouse spleen using the RNeasy Mini Kit (Qiagen GmbH, Hilden, Germany) according to the manufacturer’s instructions. The mRNA was purified using the MicroPoly(A) Purist™ small scale mRNA purification kit (Ambion, Foster City, CA, USA) and 7.8 μg was used as the template for cDNA synthesis with the Superscript™ III Reverse Transcriptase (Invitrogen, Carlsbad, CA, USA) according to the manufacturer’s protocol. Typically, 0.2 μg of mRNA was used per reaction for first-strand cDNA synthesis primed with an oligo-(dT)_20_ primer (Invitrogen, Carlsbad, CA, USA). The resulting cDNA was used as a template for the amplification of the murine IgG variable heavy (V_H_) and light (V_L_) chain gene regions as previously described (Nathan et al., 2002) incorporating flanking *SfiI* and *NotI* restriction sites in the V_L_ 5′ and V_H_ 3′ domains, respectively. All primer sequences are listed in Table S1. The primer combinations were designed to amplify most of the known mouse antibody sequences thus generating a polyclonal pool of V region fragments.

A total of 23 Vκ region PCRs (50-μl volume) were carried out to amplify the κ light chain (LC) variable region as follows: 0.4 μM MSCVκ (Vκ 5′ sense/SfiI) primer mix, 0.4 μM MSCJκ (Vκ 3′ antisense) primer mix, two μl first-strand cDNA, 0.3 mM dNTP mix (Promega, Madison, WI, USA), 1× PCR buffer and 2.0 U DyNAzyme™ II DNA polymerase (Finnzymes, Vantaa, Finland). The Vλ gene region amplifications were prepared using one set of forward and reverse primers only and were set up as above except that the primer mixes were substituted with the MSCVL-1 (Vλ 5′ sense/SfiI) and MSCJKL-B (Vλ 3′ antisense/short linker) primers. Four Vλ PCR reactions were prepared.

The V_H_ gene region was amplified using a combination of 19 forward primers and three reverse primers, generating a total of 30 V_H_ reactions prepared as described above. The V_H_ region PCR reactions included the MSCVH (V_H_ 5′ sense) and MSCG (V_H_ 3′ antisense/*NotI*) primer mixes for the amplification of V_H_ regions IgG1, IgG2a, IgG2b, IgG3, and IgM. All amplifications began at 94 °C for 3 min followed by 30 cycles of 94 °C for 30 s, 56 °C for 30 s, 72 °C for 90 s, and a final extension step at 72 °C for 2 min. The PCR products were purified using the QIAquick Gel Extraction Kit (Qiagen GmbH, Hilden, Germany).

The V_H_ and V_L_ coding sequences were fused using the flanking primers MSCF (5′-GCG GGG CCC AGC CGG CCG AGC TCG-3′) and RSCB (5′-GCC TGC GGC CGC ACT AGT GAC AGA-3′) by splice-overlap extension PCR (SOE-PCR) to produce the scFv fragments. The scFv assembly reaction comprised one μM MSCF primer, one μM RSCB primer, ∼50 ng each of the V_H_ and V_L_ coding sequences, one mM dNTP mix, 2× amplification buffer, one mM MgSO_4_, 2× PCR_x_ enhancer solution, and one U Platinum *Pfx* DNA Polymerase (Invitrogen, Carlsbad, CA, USA). The reactions comprised seven cycles at 92 °C for 1 min, 63 °C for 30 s, 58 °C for 50 s, and 72 °C for 1 min, followed by 23 cycles of pull-through reactions at 92 °C for 1 min, 63 °C for 30 s, and 72 °C for 1 min, followed by a final extension step at 72 °C for 10 min.

The resulting polyclonal pool of scFv fragments was sequentially digested with *SfiI* and *NotI* and transferred to the pCANTAB5E phagemid vector (GE Healthcare Life Sciences, Marlborough, MA, USA). The ligation products were introduced into electrocompetent *Escherichia coli* TG1 cells by electroporation (2.5 kV, 200 Ω, 25 μF, time constant >3.6 ms). Five sets of scFv electroporation transformations were carried out to generate a combinatorial scFv library in which the antibodies were expressed as fusions with the M13 minor phage coat protein pIII after rescue with a helper phage.

### Biopanning of the phage-scFv display library

The phage-scFv display library was incubated with *T. gondii* tachyzoites (2.88 × 10^4^) in a pre-blocked microcentrifuge tube at 8 °C for 2 h while rotating. The tachyzoite cells were then pelleted by centrifuging at 1,000×*g*, 4 °C, for 5 min and the supernatant was discarded. The cells were vortexed five times with Todd–Hewitt broth (THB) containing 0.05% (v/v) Tween-20 (THB-T) and centrifuged as above. Bound phage were eluted by resuspending cells in 200 μl glycine-HCl (pH 2.2) at 4 °C for 10 min and neutralized by adding 26.7 μl Tris-Cl (pH 8.0). The phage eluate was then introduced to log-phase *E. coli* TG1 cells and plated on super optimal broth with ampicillin and glucose (SOBAG) overnight at 30 °C, at 100× and 10× dilutions for phage output titering. The phage output titer was calculated from the mean of triplicate sets of each dilution to determine the number of phage captured on the cells. The remaining phage-infected *E. coli* TG1 suspension was plated on large SOBAG plates and also incubated at 30 °C overnight to obtain single colonies of *T. gondii*-binding scFv clones. Each scFv clone was verified by PCR for the presence of the full-length scFv sequence and fingerprinted with MvaI (Thermo Fisher Scientific, Waltham, MA, USA). Full-length scFv genes with unique fingerprint patterns were selected and the clones were sequenced using the dideoxy method with the pCANTAB5 sequencing primer pairs S1 and S6 (GE Healthcare Life Sciences, Marlborough, MA, USA).

### Monoclonal scFv binding titer assay

Phage rescue of the monoclonal full-length scFv clones was achieved using VCSM13 interference-resistant helper phage (Stratagene, San Diego, CA, USA). The recombinant phage suspensions for each clone (2.03 × 10^11^ p.f.u.) were blocked with 1% (w/v) bovine serum albumin (BSA) before incubation with *T. gondii* tachyzoites (1.0 × 10^5^ cells/reaction). Each scFv clone was also tested for binding to HFF cells (1.0 × 10^5^ cells/reaction) as a control. All reactions were incubated at 8 °C for 2 h while rotating. The centrifugation, washing, phage-elution steps, and re-infection steps were carried out as described above. The means of the samples and negative controls were compared using the one-tailed Wilcoxon–Mann–Whitney statistical test (*H*_0_ < *H*_A_, α = 0.05) in SigmaPlot v11.0 (Systat Software Inc., San Jose, CA, USA). The exact *p* value of the Wilcoxon Mann–Whitney statistics was computed using Analyse-it® v2.22 statistical analysis software for Microsoft Excel.

### Sequence analysis and structural modeling of V regions

The full-length V_H_ and V_L_ chain sequences were numbered according to the International ImMunoGeneTics (IMGT) unique numbering scheme (Lefranc et al., 2003) and analyzed using V-Quest (imgt.cines.fr/textes/vquest) to determine the CDRs and germline origins. Target-unrelated peptide (TUP) motifs and sequence redundancy were identified using scanner and reporter of target-unrelated peptides (SAROTUP) and MimoDB.

Structural modeling was accomplished using the web-based antibody modeling software Rosetta Antibody: Structure Prediction Server provided by the Department of Chemical and Biomolecular Engineering, Johns Hopkins University (http://antibody.graylab.jhu.edu/) (Sircar, Kim & Gray, 2009). The protocol integrates ab initio CDR H3 loop modeling and simultaneous optimization of CDR backbone dihedral angles and the relative orientation of V_L_ and V_H_. Only first rank predictions were used for each scFv model. The analysis and superimposition of scFv molecular models was generated with PyMOL for Windows.

### Production of soluble scFv antibody fragments

Candidate scFv-phages particles were used to infect *E. coli* HB2151 cells, a non-suppressor strain for the IPTG-inducible expression of antibody fragments. Soluble scFv antibodies were extracted from the bacterial periplasm as previously described (Barbas et al., 2001). Periplasmic extracts were analyzed by 12% (w/v) SDS–PAGE (Laemmli, 1970) and transferred to nitrocellulose membranes which were probed overnight at 4 °C with an anti-E-tag primary antibody (Abcam, Cambridge, UK) diluted 1:1,000 in Tris-buffered saline (TBS) with 2% (w/v) skimmed milk. Blots were washed three times with TBS-T (TBS with 0.05% (v/v) Tween-20), and incubated with a horseradish peroxidase-conjugated anti-rabbit IgG secondary antibody (CAT. No: A0545) (Sigma-Aldrich, St. Louis, MO, USA) diluted 1:1,000 in 2% (w/v) skimmed milk for 1 h with gentle agitation. After three further washes in TBS-T, the signal was detected with SuperSignal West Pico ECL substrate (Thermo Fisher Scientific, Waltham, MA, USA).

### Construction of affinity-matured scFv library

Antibody hotspot regions in the CDR were identified using V-Quest. The hotspot motif RGYW in scFv TG130 V_L_ CDR1 was chosen for the introduction of random mutations to produce an affinity-matured scFv antibody library. Completely overlapping sense and antisense DNA oligomers were designed to generate a library randomizing four nucleotides (two consecutive amino acids) in the hotspot while preventing the introduction of stop codons. The following degenerate oligomers were used, with the randomized sequences underlined and bold: pCANTAB5 E—RGYW Fwd (Sense), 5′-G GCC AGT CAG GAT GTG **VNS N**CT GCT GTA GCC-3′; pCANTAB5 E—RGYW Rev (Antisense), 5′-GGC TAC AGC AG**N SNB** CAC ATC CTG ACT GGC C 3′, (V = A or C or G, S = C or G, B = C or G or T). The affinity maturation reaction was carried out using the QuickChange Lightning Site-Directed Mutagenesis Kit (Stratagene, San Diego, CA, USA).

Before site-directed mutagenesis, oligonucleotide adaptors were generated from the degenerate oligomers to improve the reaction performance. Equimolar amounts of the degenerate oligonucleotides (pCANTAB5 E—RGYW Fwd and pCANTAB5 E—RGYW Rev) were resuspended in annealing buffer (10 mM Tris-HCl pH 7.5, 60 mM NaCl) and heated to 95 °C for 10 min before cooling to room temperature overnight to form an oligonucleotide duplex. Following the generation of the degenerate adaptors, the mutagenesis PCR reaction was carried out using 10.6 ng of the phagemid pCANTAB5E-TG130 as a template and 30.0 pmol of the degenerate DNA adaptors (15.0 pmol or 153.4 ng each of the degenerate DNA oligomers pCANTAB5 E—RGYW Fwd and pCANTAB5 E—RGYW Rev). The scFv template and DNA adaptors were mixed with the QuikChange Lightning enzyme (Agilent Technologies, Santa Clara, CA, USA) and its component reagents according to the manufacturer’s protocol in a 50-μl reaction volume and heated to 95 °C for 2 min, followed by 18 cycles at 95 °C for 20 s, 60 °C for 10 s, and 68 °C for 2.5 min, and a final extension step at 68 °C for 5 min. The mutagenesis PCR product was digested with Dpn1 to remove all parental phagemid dsDNA, precipitated with ethanol and introduced into XL-10 Gold® ultracompetent cells (Stratagene, San Diego, CA, USA) according to the manufacturer’s protocol. Transformed clones were verified by colony PCR and sequenced to check for successful sequence diversification. Sequences were aligned using the BioEdit v7.0.5.3 (Hall, 1999). Isolated plasmid DNA was introduced into chemically-competent *E. coli* TG1 cells (Tu et al., 2005) and rescued using helper phage VCSM13 (Stratagene, San Diego, CA, USA) (Barbas et al., 2001).

### Biopanning of the affinity-matured scFv library

The affinity-matured scFv library prepared by phage rescue was precipitated with polyethylene glycol and blocked with 2% (w/v) BSA (1:1 ratio) before biopanning. The recombinant phage (∼2.7 × 10^12^ cfu) from the mutated library was incubated in triplicate with *T. gondii* tachyzoites (∼4.0 × 10^5^ cells) in pre-blocked microcentrifuge tubes and the mixture was rotated at four rpm at 8 °C for 1 h, with VCSM13 helper phage negative controls. The cells were pelleted by centrifugation at 1,000×*g*, 4 °C for 5 min and the supernatant was discarded. The cells were resuspended in cold THB-T, vortexed and incubated for 5 min at 4,000 rpm, 8 °C. This washing step was repeated for 10 consecutive rounds, and bound phage were eluted by resuspending the cells in glycine-HCl (pH 2.2) as described above. After re-infection, the eluted phages were titered to determine the quantity of captured phages, and glycerol stocks of the eluate were prepared and stored at –80 °C.

Eluted recombinant phages were used to re-infect log-phase *E. coli* TG1 cells and plated on SOBAG medium. After an overnight incubation at 30 °C, 96 single colonies were picked and each clone was sequenced to characterize the captured affinity-matured scFv antibodies. The five unique affinity-matured phage-scFv clones were rescued using VCSM13 interference-resistant helper phage (Stratagene, San Diego, CA, USA) and assessed by biopanning against *T. gondii* tachyzoites. The recombinant phage suspensions from each clone (mean titer 2.03 × 10^11^ cfu/ml) were blocked in 2% (w/v) BSA before incubation with *T. gondii* tachyzoites (1.0 × 10^5^ cells per reaction) as described above.

### Conversion of scFv antibodies into chimeric human IgGs and transient expression

The V_H_ sequence from each scFv was inserted into the plant expression vectors pTRAkc-HC upstream of the Cγ region using the restriction sites *BspHI* and *NotI*, whereas the V_L_ sequence was inserted into pTRAkc-LC upstream of the Cκ region using the restriction sites *NcoI* and *NotI*. The pTRA plant expression vectors are derivatives of pPAM (GenBank: AY027531). The pTRA constructs were introduced into *Agrobacterium tumefaciens* GV3101 by electroporation (Eppendorf, Hamburg, Germany) using the standard settings of 2.5 kV, 5 ms, 200 Ω, and 25 μF, and the transformants were stored as a 50% (v/v) glycerol suspension at −80 °C. Bacterial suspensions were prepared for agroinfiltration (Piotrzkowski, Schillberg & Rasche, 2012) by diluting acetosyringone-induced bacteria to OD 1.0 with infiltration medium. Combinations of pTRAkc-HC and pTRAkc-LC transformants were mixed at a 1:1 ratio prior to infiltration. The suspensions of the five HC + LC combinations (corresponding to the five scFv variants) were co-agroinfiltrated into *N. benthamiana* leaves by standard syringe infiltration (D’Aoust et al., 2009) and plants were incubated in phytotrons for 5 days before harvesting.

Infiltrated leaves were weighed before grinding to a fine powder in liquid nitrogen with a mortar and pestle. Total soluble protein was extracted in two ml PBS containing 10 mM sodium disulfide per gram of leaf material. Cell debris was removed by filtering through Miracloth (Merck Millipore, Billerica, MA, USA) followed by centrifugation at 40,000×*g*, 20 min, 4 °C. The supernatant was adjusted to 500 mM NaCl and allowed to cool on ice for 30 min before centrifuging as above and passing through a 0.45-μm syringe filter.

The chimeric IgGs were purified using Protein A Sepharose (GE Healthcare Life Sciences, Marlborough, MA, USA) (Hehle et al., 2011) and desalted with PD-10 columns (GE Healthcare Life Sciences, Marlborough, MA, USA) using the manufacturer’s gravity protocol. Purified and desalted fractions were analyzed by reducing and non-reducing SDS–PAGE with Coomassie staining to determine antibody integrity. The extracted IgG was detected using alkaline phosphatase (AP)-conjugated human Fc-specific (Jackson ImmunoResearch Laboratories Inc., West Grove, PA, USA) and human κ LC-specific (Sigma-Aldrich, St. Louis, MO, USA) antibodies, and the signals were developed using NBT substrate. Purified IgG antibody concentrations were determined using a Qubit® 2.0 Fluorometer (Thermo Fisher Scientific, Waltham, MA, USA).

### ELISA and immunoblot analysis of anti-*T. gondii* chimeric IgG

*Toxoplasma gondii* antigen (CAT. No: EV8131) (Meridian Life Science, Memphis, TN, USA) was coated onto high-binding enzyme-linked immunosorbent assay (ELISA) plates (Dynex Technologies, Chantilly, VA, USA) at a concentration of 0.5 μg per well in PBS overnight at 4 °C. The plates were washed three times with PBS and blocked with 3% (w/v) BSA in PBS for 2 h at room temperature. HFF cell lysates and mouse ascites (CAT. No: M8273) (Sigma-Aldrich, St. Louis, MO, USA) as controls were coated at a concentration of 0.5 μg per well and processed as above. The recombinant IgGs purified from transformed plants were each diluted to a working concentration of two μg/ml in 3% (w/v) BSA and tested with the antigens for 2 h at room temperature. The ELISA plates were washed four times with PBS-T before detecting binding antibodies with HRP-conjugated anti-human Fc diluted 1:10,000 for 1 h at room temperature. The plates were washed three times with PBS-T, followed by two washes in PBS. The assays were developed with 1-Step™ Ultra TMB ELISA substrate (Thermo Fisher Scientific, Waltham, MA, USA) according to the manufacturer’s instructions. Reactions were stopped after 30 min with 2M H_2_SO_4_ and readings taken at OD_450 nm_ using a microplate reader (Tecan, Maennedorf, Switzerland).

For immunoblot analysis, *T. gondii* total antigen lysate (CAT. No: EV8131) (Meridian Life Science, Memphis, TN, USA) was either prepared by boiling in reducing sample buffer (1% (w/v) SDS, 10% (v/v) glycerol, 10 mM Tris-Cl, pH 6.8, one mM EDTA, 0.05 mg/ml bromophenol blue, 5% (v/v) β-mercaptoethanol) for 5 min, or mixed with NativePAGE™ sample buffer (Thermo Fisher Scientific, Waltham, MA, USA), before fractionation on a 12% (w/v) acrylamide gel. The gel was blotted onto a nitrocellulose membrane and blocked with 5% (w/v) BSA in TBST. The blotted membrane was cut into strips and probed with the chimeric IgGs at a concentration of one μg/ml, overnight at 4 °C. Bound IgGs on individual strips were detected using an AP-conjugated anti-human Fc antibody (Jackson ImmunoResearch Laboratories Inc., West Grove, PA, USA). Treated strips were developed with Western Blue® stabilized substrate for AP (Promega, Madison, WI, USA).

### Immunofluorescence microscopy

Acid-washed coverslips were treated with 0.01% (w/v) poly-l-lysine solution (CAT. No: P4707) (Sigma-Aldrich, St. Louis, MO, USA) for 10 min at room temperature, drained, dried, and covered with freshly-harvested parasites (approximately 10^5^ cells per coverslip). The cells were allowed to attach for 1 h at 37 °C followed by fixation in 2.5% formaldehyde for 30 min on ice. The samples were blocked with 3% (w/v) BSA in PBS. To visualize the binding of chimeric antibodies to the surface of intact parasites, fixed coverslips were incubated with each chimeric Ig antibody (five μg/ml) for 16 h at 4 °C, washed five times in PBS-T and stained with AlexaFluor 488-goat-anti-human-IgG (Jackson ImmunoResearch Laboratories Inc., West Grove, PA, USA) for 1 h. Coverslips were mounted with ProLong Gold (Thermo Fisher Scientific, Waltham, MA, USA) and cured for 24 h. All staining steps were conducted with antibodies diluted in 3% BSA in PBS. Coverslips incubated with the buffer control and secondary antibodies were included as negative controls. Results were visualized using a 100 × lens on an Olympus BX51 microscope.

### Invasion and attachment assays

Parasite invasion assays were carried out as described (Tyler & Boothroyd, 2011) with minor modifications. Briefly, washed RH parasites were incubated in DMEM with 2% (v/v) FCS supplemented with 35 μg/ml of each antibody or 1.5% volume of a buffer control (PBS) and incubated at 37 °C for 30 min while rotating. Invasion was encouraged by incubating on coverslips confluent with a monolayer of HFF cells for 2 h at 37 °C in a CO_2_ incubator (1 × 10^7^ tachyzoites in 140 μl per coverslip for six-well plates). Infected monolayers were then gently washed twice in PBS and fixed in 2.5% formaldehyde. Extracellular parasites were stained with rabbit anti-*T. gondii* polyclonal antiserum followed by AlexaFluor 594-donkey-anti-rabbit secondary antibodies. Infected monolayers were first permeabilized with 0.2% Triton X-100 in PBS and then blocked with 3% BSA. Parasites were then stained with goat anti-*T. gondii* polyclonal antiserum followed by AlexaFluor 488-donkey-anti-goat secondary antibodies. All primary and AlexaFluor-conjugated secondary antibodies were obtained commercially (Abcam, Cambridge, UK). Coverslips were mounted with Prolong Gold containing DAPI (Thermo Fisher Scientific, Waltham, MA, USA) overnight. The numbers of green (intracellular and extracellular) and red (extracellular) tachyzoites were scored on 30 randomly-selected fields on each of two separately-mounted coverslips with different antibody treatments, and visualized using a 100 × lens on an Olympus BX51 microscope. All digital images were acquired and analyzed using the Nikon NIS Elements software. Results are representative of data from at least two independent experiments.

Attachment assays proceeded as described for the invasion assay, except that the HFF monolayers on coverslips were pre-fixed with 2.5% paraformaldehyde before parasite incubation. Freshly-released tachyzoites were incubated with the monoclonal antibodies (25 μg/ml IgG in DMEM per 1.0 × 10^6^ tachyzoites) separately for 30 min at 37 °C while rotating. Pre-treated tachyzoites were then allowed to attach to fixed monolayers on a six-well plate for 15 min at 37 °C. The attached parasites were stained with rabbit anti-*T. gondii* polyclonal antiserum followed by AlexaFluor 594-donkey-anti-rabbit secondary antibodies. After washing, fixing and mounting, the number of attached parasites was determined in 15 random fields at 40× magnification. All assays were carried out in triplicate.

### Plaque assay

Tachyzoites were pre-treated at 37 °C for 30 min before infection with the individual monoclonal antibodies, each at a concentration of 2.5 μg/ml. Confluent monolayers of HFF cells were grown in six-well plates and seeded with 1.0 × 10^4^ tachyzoites per well for 2 h at 37 °C. The monolayers were washed and replenished with fresh DMEM, before further incubation for 5 days at 37 °C. The monolayers were then fixed in absolute ethanol for 5 min and stained for 1 min with a crystal violet solution as described (Martínez-Torrecuadrada et al., 2005). Images were captured using a Nikon Eclipse TS100 inverted microscope, with 20× magnification. A total of 20 random fields per coverslip were captured for each treatment and the plaque area was measured using Nikon NIS Elements software. All assays were carried out in triplicate.

## Results

### Biopanning a phage–scFv display library to isolate *T. gondii*-specific antibodies

Mice were immunized with *T. gondii* tachyzoites over a period of 3 weeks and were found to be seropositive for infection by immunoblot (Fig. S1). The mice were sacrificed to harvest the spleens, and scFv antibody fragments were constructed by amplifying V_H_ and V_L_ gene fragments from the isolated splenic mRNA and then fusing the V_H_ and V_L_ regions by SOE–PCR with an intervening seven-amino-acid flexible linker sequence (GGSSRSS) (Fig. S2). At least 10 separate rounds of PCR were carried out during the construction of the scFv fragments to increase library size and diversity.

The scFv fragments were cloned into the phagemid vector pCANTAB5E and expressed as a phage display scFv library with a resulting complexity of 1.62 × 10^4^ independent transformants. Antibodies with appropriate binding activities were selected by a single round of solution-phase biopanning with *T. gondii* tachyzoites. Multiple rounds of biopanning were undesirable because this resulted in the enrichment of mostly truncated antibody fragments with stop codons in the variable regions (Figs. S3–S7).

The polyclonal biopanned scFv library had a sixfold higher binding titer to the parasite cells than the un-panned scFv library, indicating enrichment for *T. gondii*-binding scFvs. Biopanning resulted in the selection of four different antibody fragments with *T. gondii* binding activity, namely scFvs TG64 (GenBank ID: JN104603), TG69 (GenBank ID: JN104604), TG116 (GenBank ID: JN104605), and TG130 (GenBank ID: JN104602). The corresponding amino acid sequences were analyzed using IMGT V-Quest (imgt.cines.fr/textes/vquest) (Table S2). Antibody germline alignments are provided in Fig. S8. Based on this analysis, the divergence of the selected variable domain sequences from germline counterparts is summarized in Table 1.

**Table 1:** Anti-Toxo scFv IGHV and IGKV percentage identity and somatic mutations at the nucleotide and amino acid level.

Clone ID	IGHV						IGKV					
	V-region identity (%)	V-region identity (nt/nt)	J-region identity (%)	J-region identity (nt/nt)	CDR a.a. mutations^1^ (%)	CDR a.a. mutations^2^	V-region identity (%)	V-region identity (nt/nt)	J-region identity (%)	J-region identity (nt/nt)	CDR a.a. mutations^1^ (%)	CDR a.a. mutations^2^
TG64	96.5	278/288	100.0	48/48	0.0	0	97.8	261/267	97.3	36/37	0.0	0
TG69	99.0	285/288	90.7	39/43	9.5	2^3^	99.6	269/270	100.0	35/35	0.0	0
TG116	100.0	288/288	100.0	48/48	0.0	0	98.2	265/270	97.3	36/37	11.1	2^4^
TG130	97.6	281/288	93.8	45/48	8.3	2^3^	95.9	259/270	94.6	35/37	22.2	4^3^

**Notes:**

DNA (nt) and amino acid (a.a.) sequences of recombinant scFv-phages isolated after biopanning with *T. gondii* tachyzoites were aligned and comparatively analyzed against its closest homology V-region germline counterparts. V-regions’ and J-regions’ identity levels were determined through the V-Quest program analysis; while CDR mutational events were manually identified and calculated from alignments.

1 Percentage of total number of CDR1–CDR3 a.a residues that differs from its germline a.a sequence of origin, presumably produced during the somatic hypermutation of B cells on exposure to antigens.

2 Total number of a.a residues mutated from the germline sequence of origin in CDR1–CDR3.

3 Amino acid residue mutations present in CDR3.

4 Amino acid residue mutations present in CDR1.

Sequence divergence from the germline repertoire reflects somatic hypermutation events in B cells during affinity maturation (MacLennan, 1994; Li et al., 2004) and indirectly indicates high affinity and/or efficacy. V-Quest analysis showed that scFv TG130 contained the most amino acid point mutations in the CDRs, that is, six residues in total (V_H_ and V_L_ chains), most of which were localized in CDR3 (Table 1), which usually contributes the most significant contacts in antibody–antigen interactions. In contrast, scFvs TG64, TG69, and TG116 contained fewer deviations from germline sequences (TG64 = 0, TG69 = 2, TG116 = 2) potentially indicating a lower degree of affinity maturation. The sequences were then analyzed using SAROTUP (http://immunet.cn/sarotup/) to exclude false-positive antibody fragments (Huang et al., 2010). This indicated that scFvs TG64, TG69, and TG116 (but not TG130) contained TUP motifs (Table S3).

**Figure 1:**
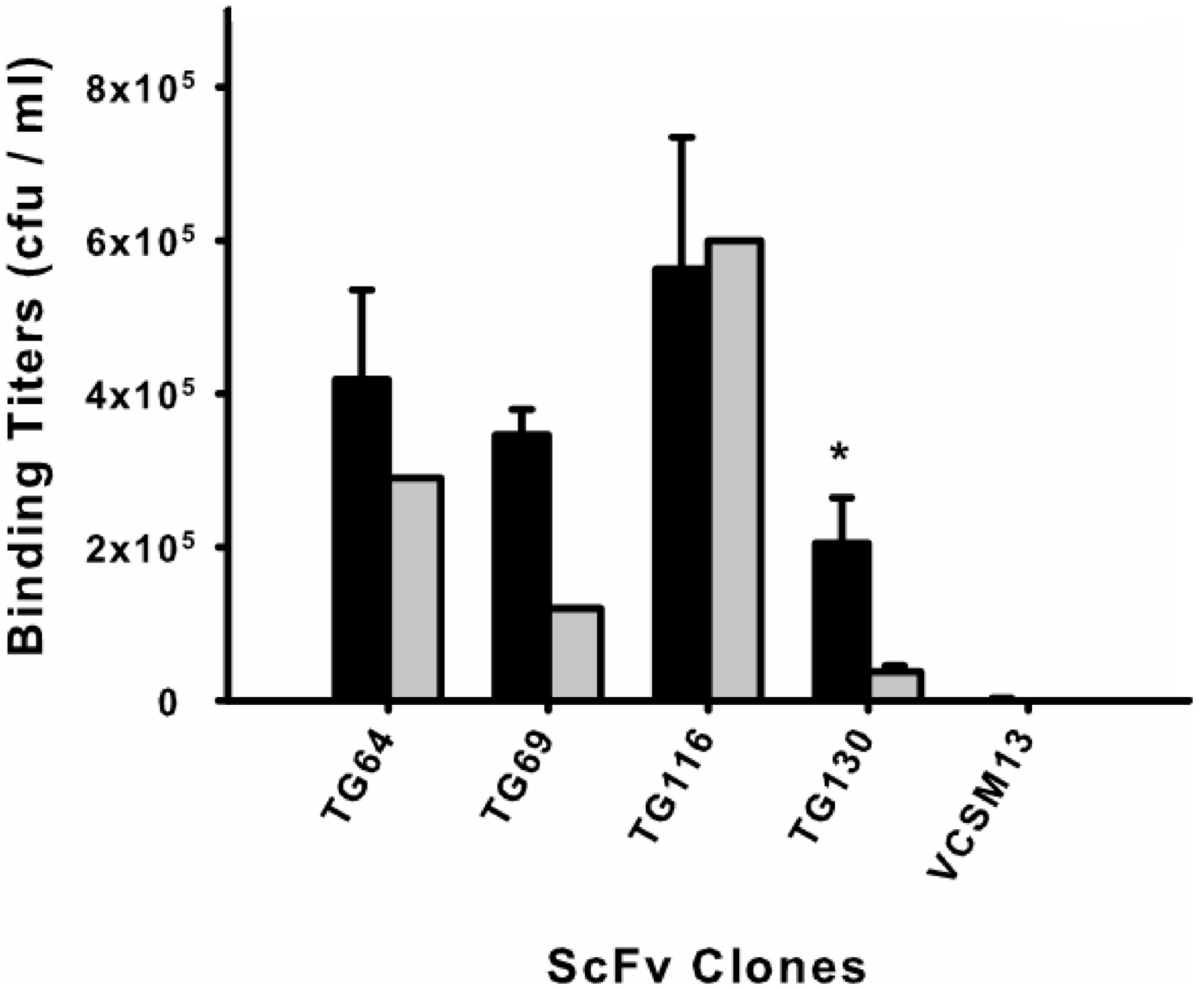
Monoclonal scFv binding titers to *T. gondii* tachyzoites and HFF. Candidate scFv antibody clones from the biopanning selection were tested to determine differential binding between the target parasite *T. gondii* (black) and a human cell line (HFF) (gray). Phage-displayed scFv clones were incubated with either parasite or HFF cells at equivalent pfu for 1 h before washing and elution. An asterisk (*) denotes a statistically significant difference in scFv binding to *T. gondii* (*p* < 0.05). Untransformed M13 phage binding titers are shown as negative controls to rule out background binding. Error bars represent standard error of the mean (SEM) for duplicate experiments.

Phage rescue was used to produce recombinant phage particles fused to the selected scFv clones for a monoclonal biopanning assay. Each of the scFv–phage clones was titered before and after biopanning (input and output titers, respectively) by eluting the scFvs from tachyzoites to determine their recovery (Fig. 1). Beginning with an average phage–scFv input titer of 10^11^ cfu/ml for each clone, we found that scFv TG116 had the highest *T. gondii* binding titer (5.62 × 10^5^ cfu/ml) and scFv TG130 the lowest (2.05 × 10^5^ cfu/ml). However, statistical analysis revealed no significant difference in the ability of three of the four isolated scFvs to bind *T. gondii* and HFF cells (*p* = 0.05, *n* = 12) despite initial promising binding titers: *P*_TG64_ = 0.44, *P*_TG69_ = 0.11, and *P*_TG116_ = 0.50), indicating that these antibodies were not specific for the parasite target.

The remaining antibody (scFv TG130) was more promising and showed significant *T. gondii* binding specificity (*p* = 0.05, *n* = 12) with a fivefold higher binding titer for the parasite compared to HFF (*P*_TG130_ = 0.0303; Fig. 1). Antibody specificity should take precedence over titers as an indication of *bona fide* antigen-specific binding. These results are consistent with our bioinformatic analysis showing that scFvs TG64, TG69, and TG116 contained TUPs, were less mature and more likely to be nonspecific. In contrast, scFv TG130 combined unique antibody CDR sequences with high target specificity, justifying its further characterization and functional validation.

### Structural analysis of scFv TG130

**Figure 2:**
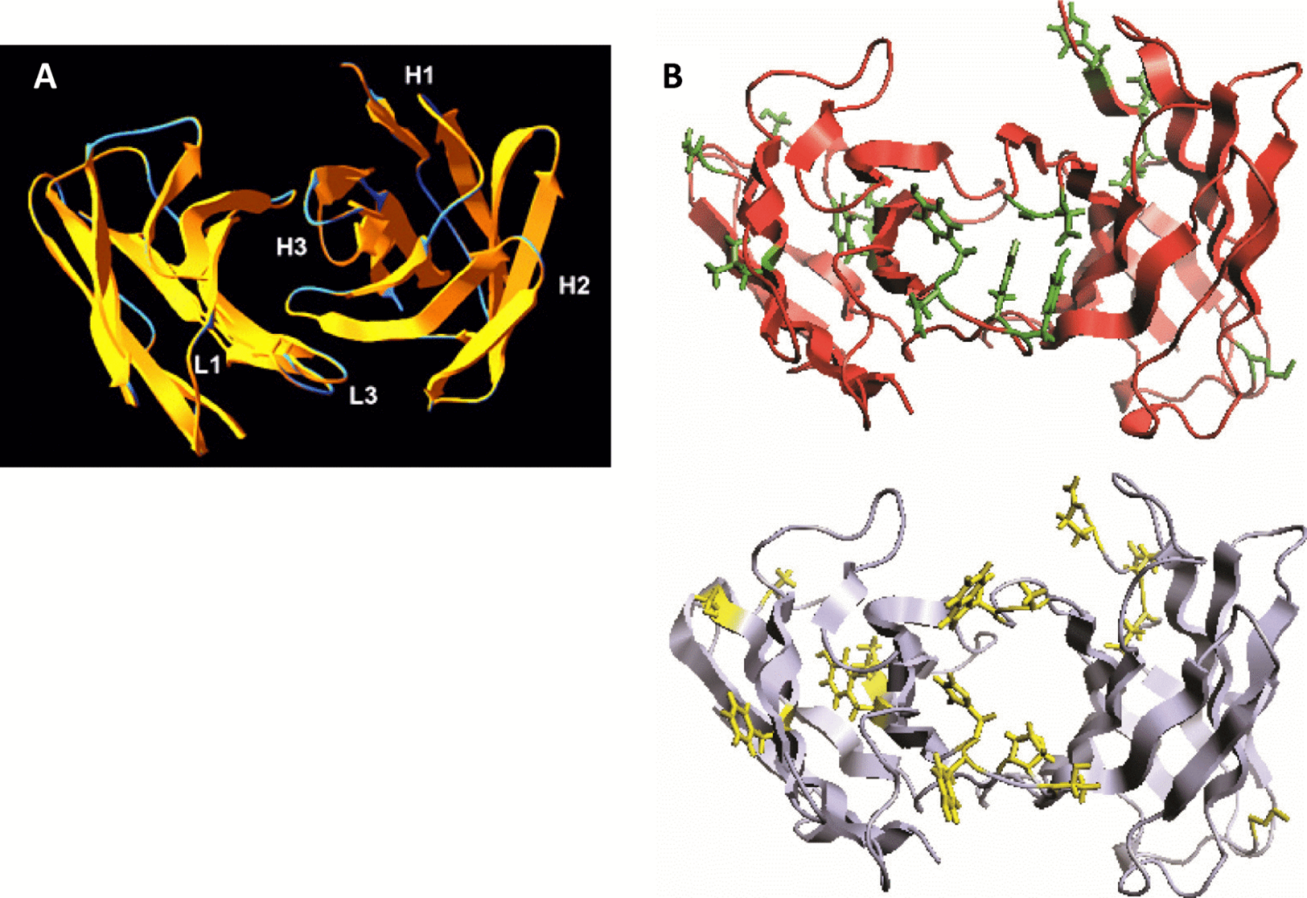
Somatic mutations and structural divergence between scFv TG130 and its germline precursor sequences. (A) Ribbon diagram of the scFv TG130 variable regions (*orange*) superimposed on its germline counterpart (*cyan*). H1, H2, and H3 = heavy chain CDR1, CDR2 and CDR3; L1 and L3 = LC CDR1 and CDR3. L2 (LC CDR2) is located on the upper right loop directly above the L1 loop. Heavy chain CDR3 (H3) for TG130 shows significant displacement from its germline counterpart structure, suggesting antibody somatic mutations arising from antigen exposure. (B) Comparison of scFv TG130 and its germline counterpart antigen-combining sites. The molecular structures of the variable regions of TG130 (red ribbons) and germline antibody (gray ribbons) are shown looking down into the antigen combining site. The amino acid changes caused by somatic mutations at the antigen binding cavity are indicated by the side chains in green (TG130) and yellow (germline).

We analyzed the structure of scFv TG130 using the Rosetta Antibody: Structure Prediction Server (Sircar, Kim & Gray, 2009) which allowed the generation of a three-dimensional molecular model for TG130 and its germline counterpart. The structural divergence of scFv TG130 from its germline counterpart was then investigated by molecular superimposition. Structural alignment of superimposed ribbon diagrams representing the germline and scFv TG130 V-regions showed significant structural changes at the antigen-combining site (Fig. 2). Although the CDR-L3 region of scFv TG130 had twice as many mutations (four substitutions) as the CDR-H3 region (two substitutions), molecular superimposition revealed that the greatest structural deviation was located within the heavy chain (HC) CDR-H3 loop rather than CDR-L3. The structural alignment showed that the greatest deviation point for the V_L_ chain was the L3 Thr^94^ germline residue, which had been mutated to Tyr^94^ in scFv TG130, with a root mean square deviation (RMSD) of 2.523 Å. In the V_H_ chain CDR H3 region, the greatest deviation point was the Ala^96^ germline residue, which was mutated to Asp^96^ in the scFv TG130 fragment, with a RMSD of 4.729 Å.

The molecular structure of TG130 indicated that six of the 15 somatic mutations relative to the germline counterpart (His → Tyr-L88 (CDR-L3), Tyr → Asn-L89 (CDR-L3), Thr → Tyr-L91 (CDR-L3), Pro → Tyr-L93 (CDR-L3), Ala → Asp-H100 (CDR-H3), and Trp → Gly-H101 (CDR-H3)), were fixed within the CDR-H3 or CDR-L3 loops, and thereby potentially involved in antigen binding. Nine other mutated residues were located in Vernier positions that do not make direct contact with the antigen. There was a higher percentage of amino acid residue changes within the CDRs compared to the framework regions, with 14.3% of mutations within the CDRs and 5.1% within the framework regions.

### Additional in vitro affinity maturation of scFv TG130

In order to further improve the affinity of scFv TG130 for *T. gondii*, the RGYW hotspots motif (R = A or G, Y = C or T, W = T or A) was modified by in vitro site-directed mutagenesis. The resulting affinity-matured antibody library had a complexity of 4.0 × 10^4^ independent clones. The affinity-matured library was subjected to a further round of biopanning and 96 antibody clones eluted from the parasite cells yielded four distinct clones selected for further characterization, with Arg-Ala (RA15), Arg-Thr (RT51), Arg-Ser (RS55), and Leu-Thr (LT3) substitutions at positions 27 and 28 of V_L_ CDR1 (Table 2). Multiple codon usage was also observed for each of these clones, suggesting strong selection for the specific replacement residues. RA15, RT51, RS55, LT3, and the parental scFv TG130 were selected for the generation of chimeric IgG antibodies.

**Table 2:** Sequences of the RGYW-mutant scFv obtained after parasite selection.

ScFv ID	Nucleotide sequences	Amino acid mutations	
Parental Ab—TG130	**AGT A**ct	Ser Thr	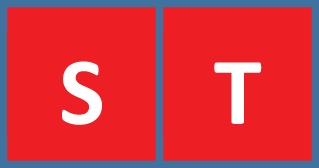
Alanine mutant—RA15	**AGG G**ct	Arg Ala	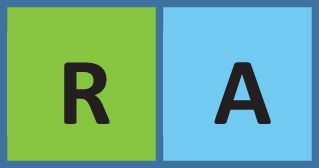
	**CGC G**ct		
	**CGG G**ct		
RT51	**AGG A**ct	Arg Thr	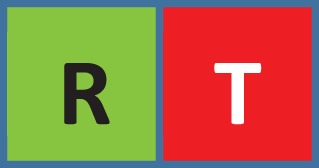
	**CGC A**ct		
RS55	**AGG T**ct	Arg Ser	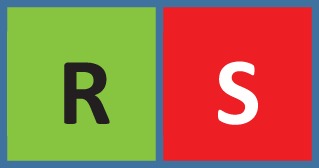
	**CGG T**ct		
LT3	**CTG A**ct	Leu Thr	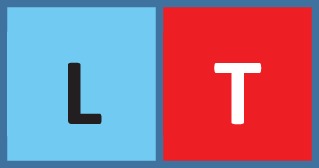
	**CTC A**ct		

**Note:**

Affinity maturation of scFv TG130 at RGYW hot spot motifs resulted in site-directed mutagenesis at amino acid positions 27 and 28 of V_L_ CDR1. The unique sequences of the mutant scFv antibodies isolated from biopanning selection against *T. gondii* are listed, along with the parental antibody. The mutated amino acid residues are color-coded according to its side-chain properties—Uncharged Polar (Red), Hydrophobic (Blue), Charged Polar (Green).

### Generation and transient expression of *T. gondii*-specific chimeric IgGs

**Figure 3:**
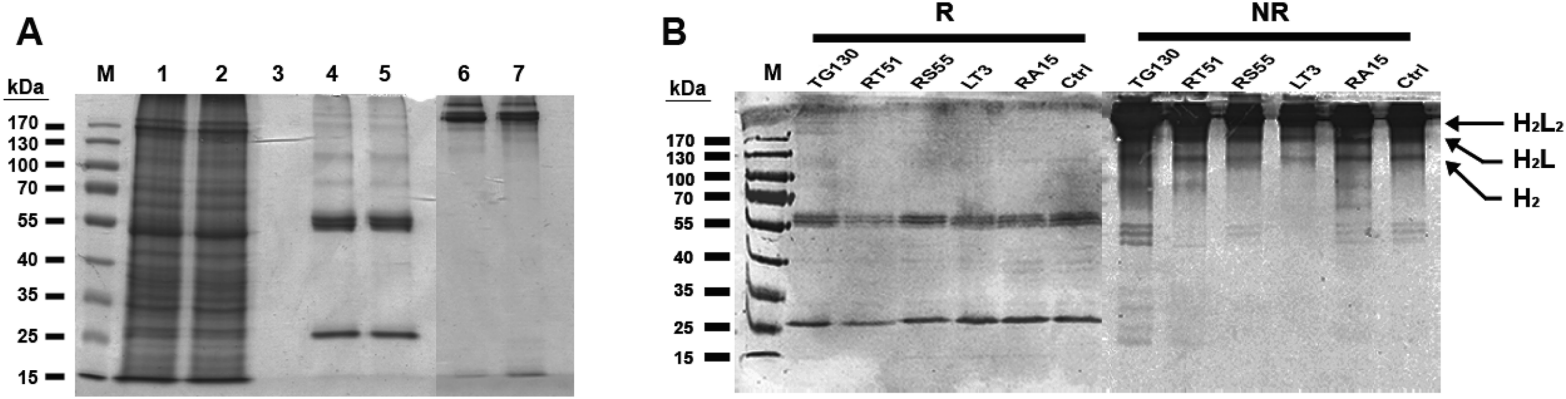
Purification of chimeric IgG antibodies transiently expressed in *N. benthamiana* leaves. (A) SDS–PAGE analysis of protein samples from the Protein A purification process: crude load (lane 1), flow fraction (lane 2), wash fraction (lane 3), first and final eluted fractions (lanes 4 and 5). Both the heavy and light chains are visible (60 and 25 kDa, respectively), whereas both eluted fractions under non-denaturing conditions show intact immunoglobulins of 170 kDa (lanes 6 and 7). (B) Immunoblot analysis of recombinant antibodies, with the designation of clones. Immunoblots of purified antibodies separated under reducing (R) and non-reducing (NR) conditions are shown, with major bands of the deduced antibody structures indicated. H_2_L_2_, IgG heterotetramer containing two HCs and two LCs; H_2_L, heterotrimer containing two HCs and one LC; H_2_, HC homodimer.

Chimeric IgG antibodies were constructed by transferring the V_H_ and V_L_ regions from the parental scFv (TG130) and the four affinity-matured derivatives (RA15, RT51, RS55, and LT3) into the binary plant expression vectors pTRAkc-HC and pTRAkc-LC, containing the human Cγ and Cκ domains, respectively. *A. tumefaciens* cultures separately transformed with the HC and LC constructs were co-infiltrated into *N. benthamiana* leaves to produce full-length IgG molecules (Fig. 3). Subsequent purification by Protein A affinity chromatography produced 32.8–72.1 μg of purified antibody per gram infiltrated fresh leaf tissue, specifically 48.6 μg/g TG130, 32.8 μg/g RA15, 45.9 μg/g RT51, 64.9 μg/g RS55, and 72.1 μg/g LT3.

The resulting antibody preparations had an average of 93% purity as estimated from SDS–PAGE and gel densitometry using Image J. Under reducing conditions, both the antibody’s HCs and LCs are visible in the eluate lane at the expected sizes of 25 and 60 kDa, respectively. With non-reducing conditions, the antibody appeared as a higher molecular weight band with the size of approximately 170 kDa, consistent with the tetrameric form of an intact IgG. Along with this tetrameric IgG, several more multimeric antibody bands were observed on the immunoblots, revealing minor degradation of the antibodies. However, the intact tetrameric IgG form showed a stronger band intensity and is validated by analytical HPLC to be the major species.

**Figure 4:**
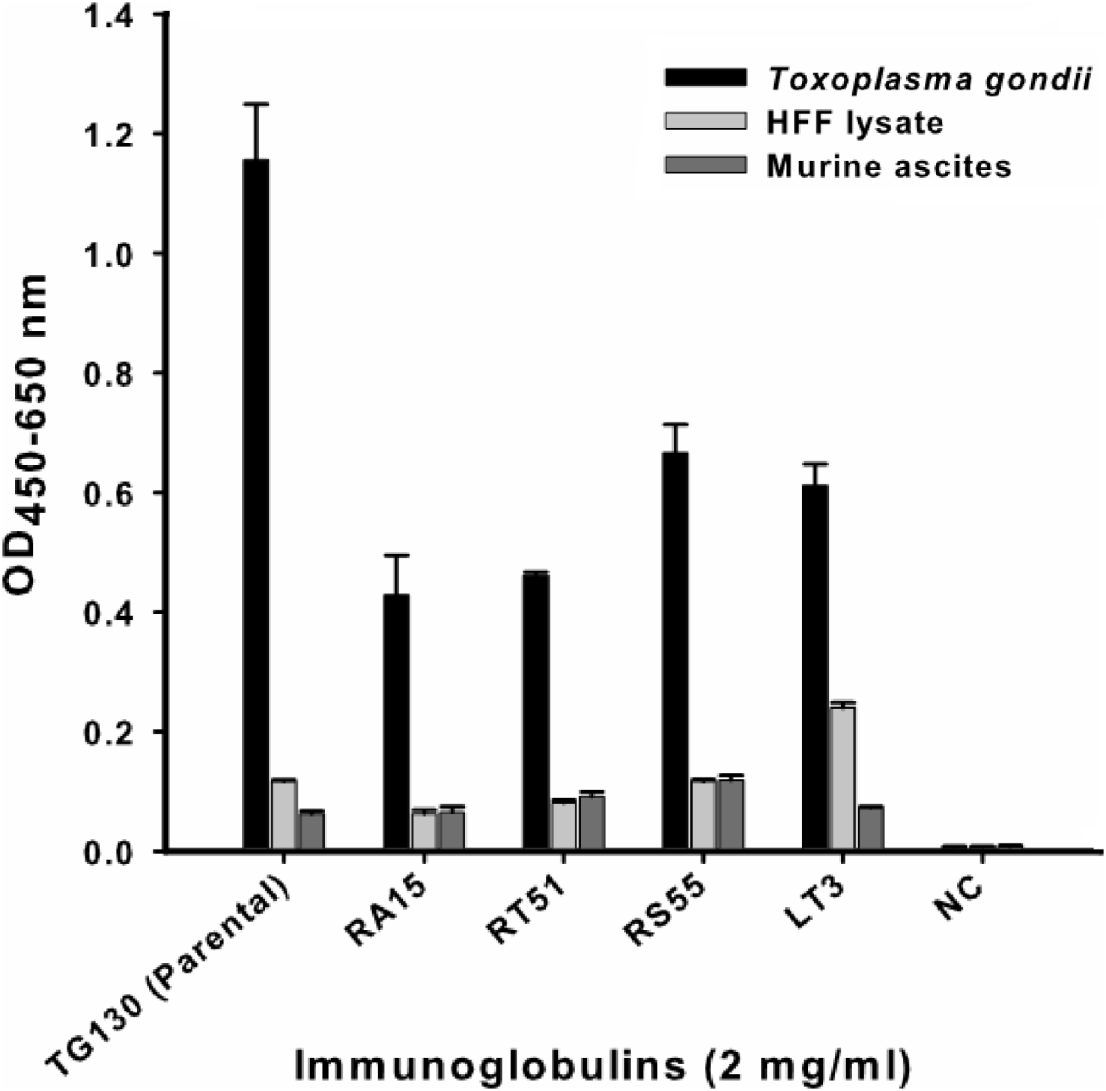
ELISA of chimeric *T. gondii*-specific IgGs. The specificity of chimeric antibodies TG130 (parental), RA15, RT51, RS55, and LT3 against lysed *T. gondii* antigen is shown by ELISA, with parallel testing against HFF lysate and murine ascites as controls. ELISA plates were coated with 10 μg/ml of each antigen and control. The HRP-conjugated secondary antibody was included as an internal negative control antibody (NC). Error bars indicate standard deviations from the means of triplicate experiments.

An indirect ELISA was used to quantify the binding of each recombinant antibody to *T. gondii* lysates, with detergent-solubilized HFF lysate and control mouse ascites (standard commercial preparation) incorporated as non-specific controls (Fig. 4). All five recombinant IgG showed significant binding to *T. gondii* compared to the control antigens. However, contrary to our expectations, the parental antibody IgG1 showed higher binding activity than the variants selected from the affinity-matured antibody library.

**Figure 5:**
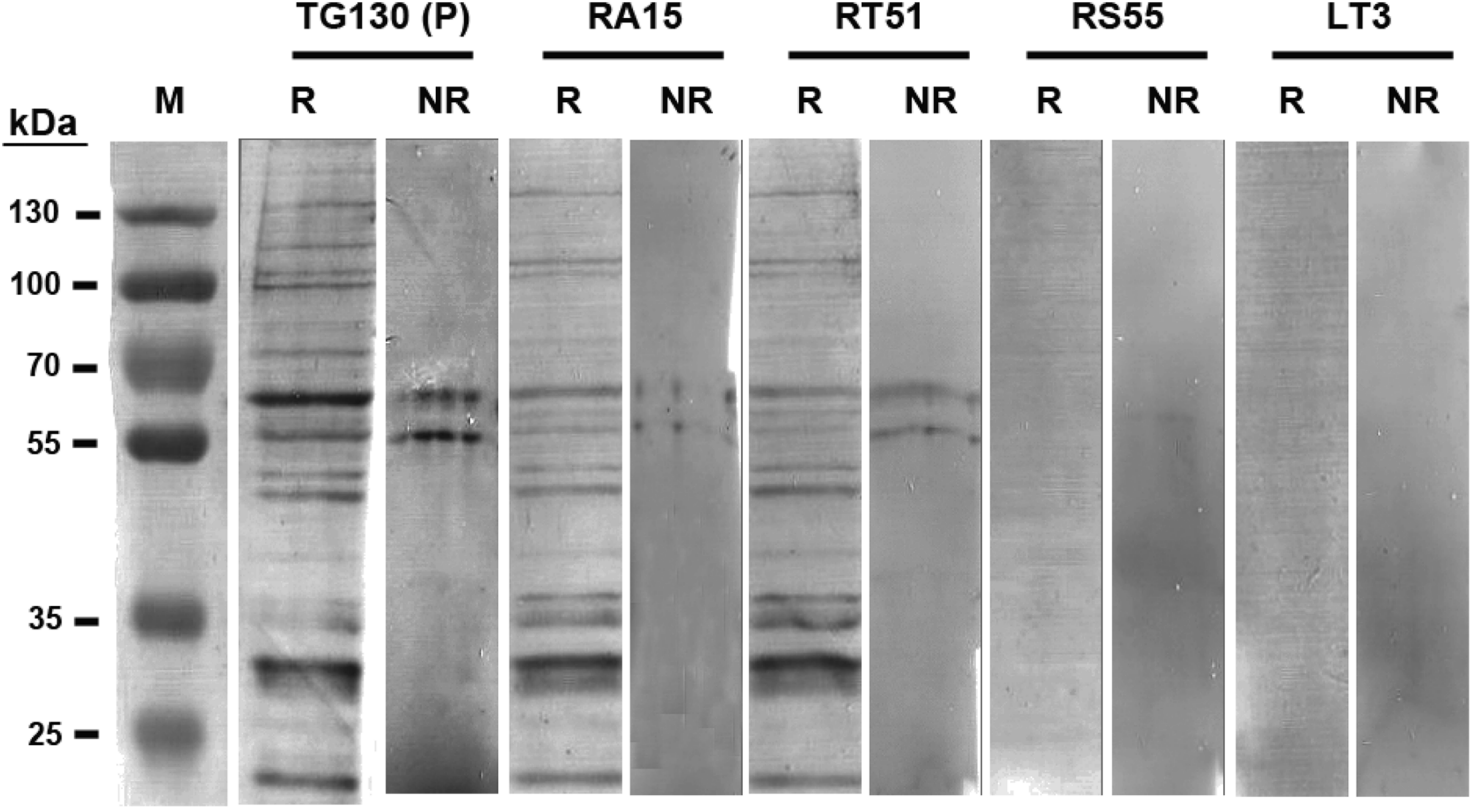
Immunoblot analysis of chimeric *T. gondii*-specific IgGs. Immunoblots of *T. gondii* lysed antigen separated under reducing (R) and non-reducing (NR) conditions, with the recombinant antibodies used for detection indicated for each blot. We used 9.6 and 3.2 μg of the *T. gondii* antigen in each lane of the reducing and non-reducing blots, respectively. The blots were probed with one μg/ml for each antibody, and an anti-Human Fc secondary antibody was used for detection followed by signal development with BCIP/NBT.

The antibodies were characterized in more detail by immunoblot analysis using both reduced and non-reduced fractions of *T. gondii* lysate (Fig. 5). We found that IgG TG130 (parental), RT51 and RA15 showed detectable binding to the parasite antigens in both the reduced and non-reduced fractions, whereas IgG RS55 and IgG LT3 consistently showed no detectable binding to any fractions. The lysates were fractionated by reducing SDS–PAGE and the three positive antibodies (parental TG130 plus RT51 and RA15) highlighted multiple bands, the most prominent of represented antigens of ∼60 and ∼30 kDa. Under non-reducing conditions, the same antibodies detected antigens of ∼55 and ∼60 kDa. IgG RA15 generated particularly weak binding signals.

**Figure 6:**
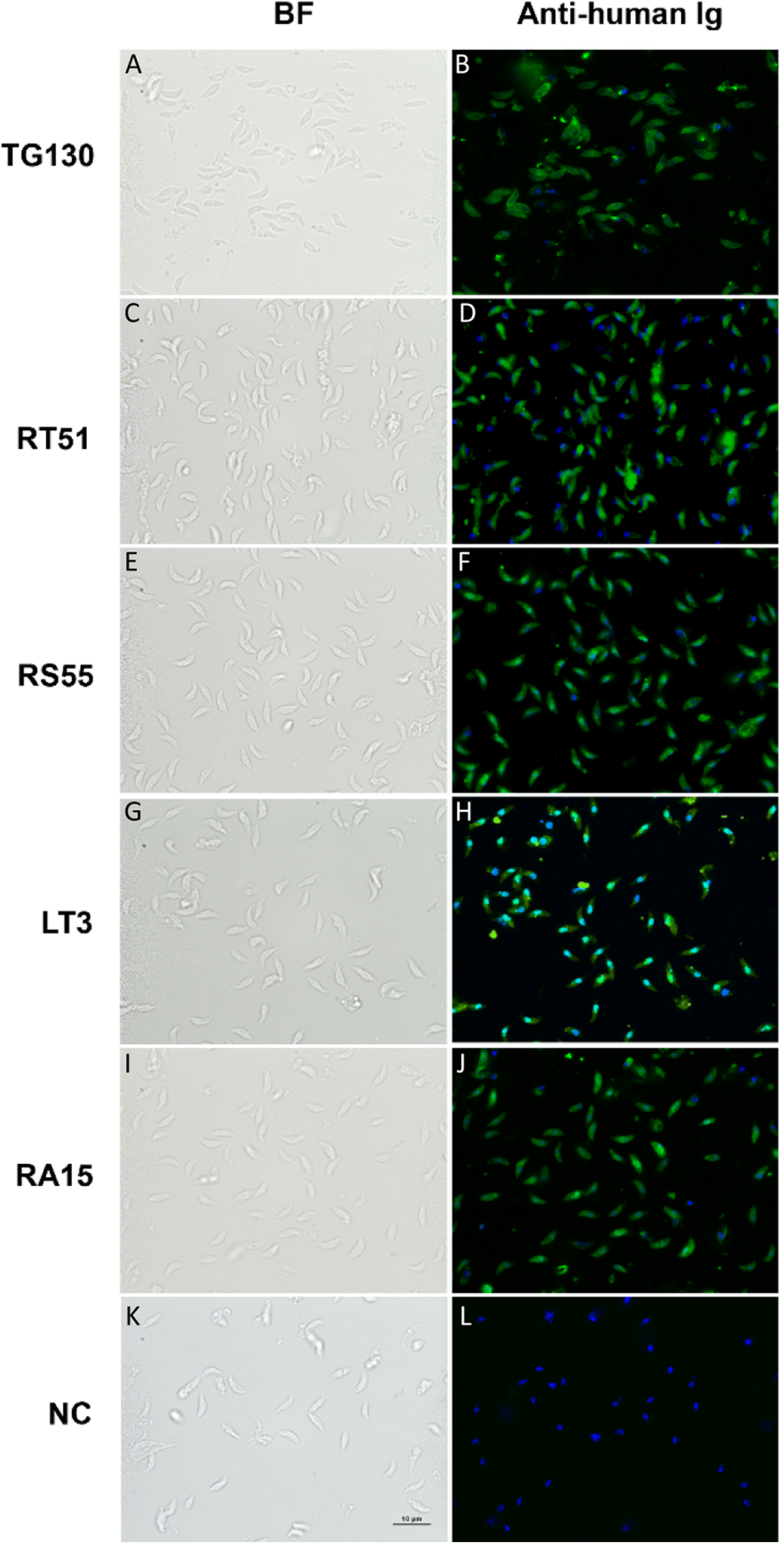
Visualization of chimeric Ig binding to the parasite surface. Immunofluorescence assays were used to detect extracellular parasites immobilized on poly-lysine coverslips to confirm the binding of each chimeric antibody (A–J) and buffer control (NC; K and L). Parasites pre-treated with the antibodies or buffer control were fixed in formaldehyde and stained in the absence of permeabilization. The chimeric antibodies bound specifically to the parasite surface as shown by the bright staining of the whole parasite, whereas the buffer control showed no binding. Scale bar = 5 μm.

Immunofluorescence assays (IFAs) were carried out to visualize the binding of the chimeric antibodies to parasite surface proteins in the native context (Fig. 6). Despite the variable results obtained in the ELISA and immunoblot experiments, we observed specific immunostaining for all IgGs to the surface of *T. gondii* tachyzoites (Fig. 6). This ambiguity across different immunoassays may reflect differences in the abundance and the folding of the parasite antigens. We used lysed, detergent-solubilized parasite antigens for the ELISA and immunoblots, but native antigens present on intact cells for the IFAs. The negative controls yielded negligible binding signals in all experiments as anticipated.

### Inhibition of parasite invasion by recombinant IgGs

**Figure 7:**
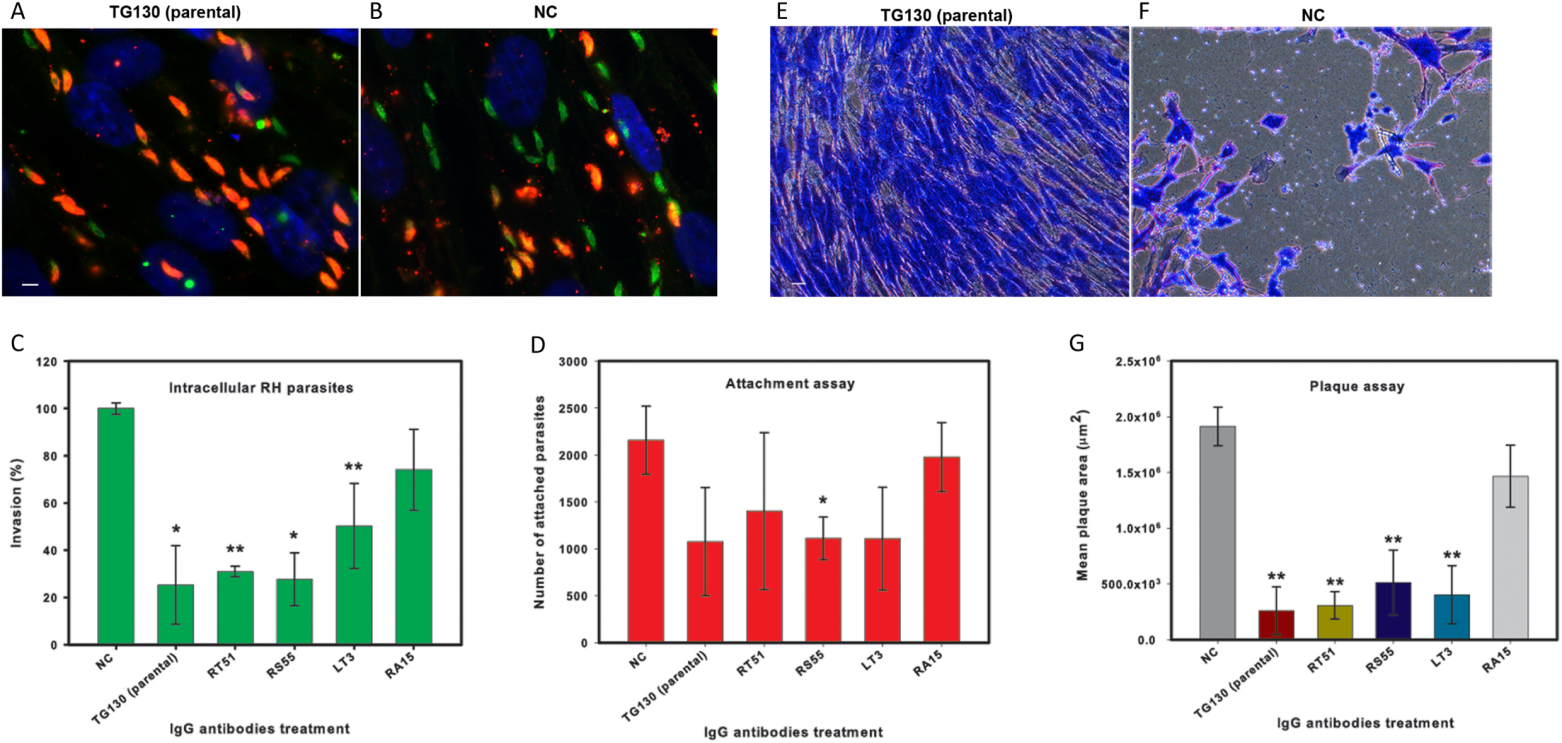
IgG treatment of *T. gondii* parasites inhibits invasion. Freshly harvested parasites (1 × 10^7^) were pretreated with buffer control (NC) or 35 μg/ml of each chimeric antibody and then allowed to infect HFF monolayers for 2 h under normal culture conditions. The number of invasive parasites was determined using differential staining of the extracellular (red, red + green) and intracellular parasites (green) before and after detergent permeabilization. (A and B) Invasive efficacy of NC and IgG-treated parasites. Immunofluorescence microscopy showed a significant decrease in the number of invasive parasites (green) with more parasites remaining outside the cell (red, red + green) compared to NC. (C) The number of intracellular parasites was scored for 30 randomly-selected fields from two coverslips for each IgG tested and NC. (D) Tachyzoites attached to paraformaldehyde-fixed fibroblasts after incubation with the chimeric IgG and were compared to the NC. (E and F) Plaque assays of HFF monolayers infected with tachyzoites treated with plant-derived IgG antibodies (2.5 μg/ml), compared with tachyzoites treated with NC that were allowed to grow for 5 days. (G) The size of the lysis plaques was measured. Invasion levels following each antibody treatment are shown as mean percentages relative to NC ± standard deviation. Parasite attachment to host cells was scored for each antibody treatment and shown as a mean score ± standard deviation. The invasion assay was carried out in duplicate, whereas the attachment and plaque assays were carried out in triplicate. An asterisk or double asterisk denotes a statistically significant reduction in parasite invasion relative to NT (Student’s *t*-test, *p < 0.05; **p < 0.01).

Finally, we investigated the ability of the chimeric antibodies to inhibit the invasion of human fibroblasts using the red/green invasion assay (Figs. 7A and 7B). We found that three of the chimeric IgGs caused a significant decline in parasite invasion efficacy relative to untreated controls, namely 75% for parental TG130, 69% for RT51, and 72% for RS55, whereas IgG RA15 showed no significant inhibition of invasion (Fig. 7C).

In order to determine whether the inhibition of parasite invasion reflected an impairment of parasite binding to host cells, we measured the ability of IgG-treated parasites to bind host cells fixed in paraformaldehyde, which renders them more rigid and resistant to invasion. With the exception of RA15, all treated parasites showed a partial (35–67%) reduction in host cell attachment compared to the untreated controls, but the attachment scores differed significantly only for IgG RS55 (48.5% inhibition) as shown in Fig. 7D. IgG RA15 showed little improvement in the ability to block parasite attachment compared to the untreated control (8.3% inhibition).

A plaque assay was used for further validation of the effect of chimeric IgGs on invasion, because this assay recapitulates all the steps of the lytic cycle. Remarkably, parental IgG TG130 and RT51 achieved an 87% reduction in mean plaque area compared to untreated controls (Fig. 7G). The parasites treated with these antibodies formed substantially smaller plaques compared to controls even at a low IgG concentration of 2.5 μg/ml (Figs. 7E and 7F). Collectively, these results demonstrate that plant-derived IgGs, particularly TG130 (parental), and RT51 are highly efficacious ligands to inhibit the invasion of host cells by *T. gondii* in vitro.

## Discussion

The current study demonstrated the development and evaluation of five mouse-human chimeric IgGs based on a selected mutated *T. gondii* tachyzoite-specific scFv sequences. The chimeric IgGs were expressed in *N. benthamiana* plants. These IgGs were shown to bind specifically to *T. gondii* tachyzoites and inhibited the invasion of human fibroblasts, suggesting a great pharmaceutical potential of these plant-derived antibodies against toxoplasmosis.

*Toxoplasma gondii* is an opportunistic pathogen that can cause severe or lethal infections in individuals with reduced cellular immunity, for example, infants, neonates, and immunocompromised adults. The humoral and cellular immune responses must act in concert for effective resistance to toxoplasmosis. Mice deficient in B cells are more susceptible to lethal toxoplasmosis during the chronic infection phase mainly due to unrestricted parasite proliferation in the brain and lungs, causing widespread tissue necrosis (Kang, Remington & Suzuki, 2000). Although T cells and IFNγ are important mediators of host immune responses to *T. gondii*, specific antibodies produced by B cells are essential for long-term resistance (Kang, Remington & Suzuki, 2000). Therefore, neutralizing antibodies are an important therapeutic option for the disease, as has recently been demonstrated for West Nile virus infections (Oliphant et al., 2005) and Ebola virus disease (Pettitt et al., 2013).

Specific antibodies against *T. gondii* surface proteins and living cells have shown a strong potential for inhibitory activity in vitro and in vivo (Carruthers, Giddings & Sibley, 1999; Hehl et al., 2000; Liu et al., 2006; Fu et al., 2011). We therefore panned a phage-scFv library against living *T. gondii* tachyzoites and used stringent selection combined with bioinformatics analysis to isolate a promising parasite-specific scFv. The specific scFv was isolated from a relatively small scFv library (1.6 × 10^4^ independent transformants) which was generated from a single ligation reaction. Generation of a larger scFv library to the magnitude of 10^7^ independent transformants could possibly yield more specific scFv antibodies; which would require approximately ten ligation reactions followed by electrotransformation (Barbas et al., 2001).

The scFv antibody format is ideal for phage display-based screening procedures, but they are monovalent and tend to be unstable (Kupper et al., 2005). We therefore converted the scFv antibody into a full-length IgG, which was produced by transient expression in *N. benthamiana* plants. We also attempted in vitro affinity maturation by V_L_-CDR3 site-directed mutagenesis, but the detailed functional analysis of the mutant variants demonstrated that no significant improvement was achieved over the performance of the parental antibody using this approach. All mutants were able to bind specifically to *T. gondii* tachyocytes with no cross-reactivity to human cells or murine ascites. Two variants (RT51 and LT3) showed similar efficacy in parasite invasion assays compared to the parental IgG TG130, but the in vitro performance of variant RA15 was poor. The lack of antibody improvement achieved through the affinity maturation strategy could be due to the inherent limitation of low signal-to-noise ratios as equilibrium selection methods require antigen levels in the range of the antibody K_D_. The use of tachyzoite cells as native antigens was also likely to contribute to the high noise in the selection phase owing to the complex cell surface landscape. Similar work in the future to improve antibody affinity should consider the use of more nonstochastic methodology such as look-through mutagenesis which systematically generates single amino acid substitutions at each CDR positions; and computational design to predict binding affinity by calculation of binding free-energy of antibody models before generating the antibody mutants. These methods, combined with the use of competitive binding strategy in the selection phase could enable the enrichment for clones with slower-disassociation kinetics than the parent antibody and result in improved affinity.

Phage–scFv display libraries are often subjected to multiple rounds of selection on purified antigens (Ridgway et al., 1999; Giordano et al., 2001; Hof et al., 2006; Eisenhardt et al., 2007). However, we found that multiple rounds of biopanning on *T. gondii* cells resulted in the recovery of predominantly truncated proteins instead of full-length scFvs, as previously reported for other antigens (Yan, Ko & Qi, 2004; Liu et al., 2005; Martínez-Torrecuadrada et al., 2005) including the selection of Fab antibody fragments against the *T. gondii* protein SAG1 (Fu et al., 2011). Whole-cell biopanning can be challenging because heterogeneous and sticky cell surfaces tend to trap nonspecific binders, a problem compounded by the propagation of these nonspecific binders in subsequent amplification rounds (Kramer et al., 2003). However, this is offset by the advantage of screening native antigen conformations including carbohydrate epitopes in the context of complex cell-surface landscape (Siegel, 2001). Therefore, we used a single round of selection on tachyzoites resulting in the recovery of functional full-length scFv antibodies, as previously used to isolate functional antibodies against botulinum toxin (Chahboun et al., 2011).

The recovery of several nonspecific scFv antibodies during the initial screen highlights the propensity for false positives to be selected along with true target binders (Vodnik et al., 2011). We therefore carried out extensive bioinformatics analysis on the putative *T. gondii*-binding scFv sequences to rule out false positives and immature antibodies similar to germline antibody sequences (Vodnik et al., 2011). The antibody sequences were screened against the SAROTUP and MimoDB databases to exclude clones with TUP motifs and identical peptides selected on different targets, which would indicate nonspecific binding. We found that scFv TG130 and its affinity-matured counterparts lacked known TUP motifs and contained no identical peptide hits in the MimoDB database, making them the most likely candidates to show specific high-affinity binding to *T. gondii* and low cross reactivity. The molecular structure of TG130 revealed more changes of amino acid residue in the CDRs than the framework regions (14.3% and 5.1%, respectively). This is in close agreement with observed somatic hypermutation trends during the humoral response, whereby average mutational probabilities for antigen-combining site and core residues are ∼12% and ∼4%, respectively (Clark et al., 2006). Somatic mutations in the variable regions provide increased ligand diversity and are also an established hallmark of in vivo affinity maturation resulting from antigen exposure (Tonegawa, 1983; French, Laskov & Scharff, 1989). As such, the significant sequence and structural changes found within scFv TG130 indicate an antigen-driven selective process during antibody maturation from its germline precursors.

Analysis of the variable domain sequences revealed that the closest murine germline sequence matching the scFv TG130 V_H_ gene was IgHV1S29*02 in the VH1 family and JH3 for the J segment. Both VH1 (together with VH5) and JH3 are germline elements that correlate with protective immunity in parasite infections (McCoy et al., 2008), for example, VH1 and JH1 are found in murine inhibitory antibodies against *T. gondii* (Chen et al., 2003) and VH5 and JH3 are found in murine inhibitory antibodies against *Plasmodium* spp. (Igonet et al., 2007). The closest murine germline sequence matching the scFv TG130 V_L_ gene was IgKV6-17*01 in the Vκ6 family and Jκ5 for the J segment. Unlike the predominance of VH1 and VH5 genes in the HC repertoire of antibodies involved in parasitic infections, there is a weak bias towards the Vκ4 family in the LC (Igonet et al., 2007; Lazarou et al., 2009). A protective antibody against *P. falciparum* (*Pf*MSP1 antigen) comprised the same V_H_ (VH1) and V_L_ (Vκ6) repertoire as scFv TG130 (Lazarou et al., 2009).

The V_H_ region of scFv TG130 showed 94.8% identity to the germline sequence at the amino acid level, typical of an affinity-matured antigen-driven immune response. This agrees with several previous phage-antibody display studies (scFv and IgG) showing comparable levels of somatic hypermutation. For example, several cancer-targeting antibodies have been described with 93–94% identity to germline sequences in the V_H_ region (Hansen, Nielsen & Ditzel, 2002; Ho et al., 2005), and virus-targeting antibodies have been described with 92.8–99.0% identity (David et al., 1995; Ray et al., 2001). A similar trend can also be observed for the V_L_ regions in these antibodies.

RGYW hotspot motifs are DNA sequences that are frequently mutated during in vivo affinity maturation (Neuberger & Milstein, 1995; Dörner et al., 1998). V-Quest analysis of scFv TG130 (Lefranc, 2001) revealed a total of five RGYW hotspots within the variable regions, all located within the CDRs (Fig. S9). Hotspots are found either in the germline sequences or close to non-germline sequences, but we focused on germline sequences because previous studies have shown they are more suitable for affinity maturation in vitro (Ho et al., 2005). We chose the germline RGYW hotspot sequence within V_L_ CDR1 for a preliminary round of affinity maturation. A pair of degenerate primers spanning the target RGYW sequences was designed to introduce random paired amino acid substitutions but not stop codons, thus avoiding the generation of truncated and non-functional antibodies.

The binding specificity of scFv antibodies converted into IgGs was demonstrated by ELISA which revealed significantly higher binding affinity for the target parasite compared to negative control antigens. However, the characterization of the IgGs showed that the affinity-matured IgGs did not confer greater binding affinity for the parasite antigen compared to the parental IgG. To avoid this issue, incorporation of an ELISA test of mutated scFvs to help inform selection of higher affinity antibodies as candidates for IgG conversion should be considered in similar work. We found that immunoblotting data was not completely commensurate with the strong ELISA signals. Of five recombinant IgG antibodies tested, only two (parental TG130 and RT51) showed clearly positive immunoblot signals, revealing that one or two substitutions in the CDR can result in a conspicuous difference in antibody binding characteristics. This is expected as mutagenesis often occurs on hotspot residues, which serve as a ligand binding site. Immunoblot analysis using fractionated, non-reduced parasite lysate showed that all two positive antibodies bound to antigens of ∼55 and ∼60 kDa, suggesting that the same epitope was recognized by the parent antibody and derivatives. The reducing and non-reducing immunoblots (Fig. 5) showed different banding patterns, with the non-reduced blots showing fewer bands compared to the reduced blots—indicative of the parasite’s multimeric proteins with its disulfide bonds intact. As has also been shown in another study, an immunoblot analysis of *T. gondii* parasite lysate under reduced conditions would tend to result in multiple non-specific bandings, with a more prominent banding of the protein-of-interest (Besteiro et al., 2009). Therefore, it was important to run an immunoblot analysis under both reduced and non-reduced conditions to gain better clarity on the specificity of the antibodies.

Interestingly, the affinity-matured IgGs bound with 42–63% lower efficiency to *T. gondii* lysates than the parental antibody despite showing the same specificity. Overall, our results showed that paired amino acid substitutions within the V_L_ CDR1 had a significant impact on ELISA and immunoblot performance but these changes were better tolerated in IFA experiments. Sequence bias in the affinity-matured antibody library can have this effect but is unlikely in our case because an evaluation of sequence diversity showed a near-even sequence distribution at the target loci (Table S4). However, it is possible that above an affinity threshold, other factors can affect the formation of an antibody-antigen complex, as documented for several cancer-targeting antibodies (Nielsen et al., 2000). This is supported by the lack of correlation between antibody affinity, specificity, and functionality (Nap et al., 1992), with instances where lower-affinity antibodies have shown better targeting and retention properties (Nielsen et al., 2000; Rudnick et al., 2011; Yu et al., 2011; Bien-Ly et al., 2014). These findings confirm that the functionality of antibodies is influenced by many different factors, and we are currently investigating the context of such interactions by identifying the parasitic epitopes involved.

We also found that treating tachyzoites with the plant-derived IgGs inhibited the invasion of host cells by up to 75%. Murine polyclonal antibodies against *T. gondii* surface antigens P30 and AMA1 were previously shown to inhibit invasion by 87% and 40%, respectively (Mineo et al., 1993; Hehl et al., 2000), whereas a murine monoclonal IgG raised against a *P. falciparum* erythrocyte-invasion antigen achieved a 38% inhibition of invasion (Triglia et al., 2011). The monoclonal IgGs in this study therefore achieved a potent inhibition of invasion comparable to the levels afforded by polyclonal antisera. More remarkably, our experiments showed that the plant-derived chimeric IgGs also inhibited plaque formation by *T. gondii* on HFF monolayers by 87% (TG130 and RT51) even at a low antibody dose of 2.5 μg/ml. The individual attachment data sets showed substantial variability between the experiments but all IgG variants except RA15 demonstrated positive blocking activity. We therefore conclude that defective parasite attachment to host cells may not be the main inhibitory mechanism but it plays a substantial contributory role. Based on these encouraging findings, we are currently investigating the in vivo efficacy of our antibodies during toxoplasmosis infections. Taken together, our results demonstrate that plant-derived monoclonal antibodies can substantially inhibit the in vitro activity of *T. gondii* and, if these results can be confirmed in vivo, the antibodies could be developed into a therapeutic option for immunocompromised patients suffering from toxoplasmosis.

Antibody-based pharmaceuticals are one of the fastest-growing market segments in the biopharmaceutical industry, but the deployment of antibodies against infectious diseases is restricted by high production costs. Plants can address this issue by reducing the cost of antibody production by up to 90% compared to fermenter-based manufacturing (Daniell, Streatfield & Wycoff, 2001; Beasley, 2014), as well as offering further advantages in terms of safety and scalability (Stoger et al., 2005). We chose *N. benthamiana* as the production platform for our antibodies because the tobacco family is well-established as a host for recombinant protein expression and is recognized by the regulatory bodies governing the production of biopharmaceuticals (Schillberg, Fischer & Emans, 2003; Stoger et al., 2005). Tobacco plants can produce high antibody yields, including a tumor-specific full-length human IgG at 500 mg/kg fresh biomass (Giritch et al., 2006; Frenzel, Hust & Schirrmann, 2013) and up to 600 mg/kg for a scFv (Rosenberg et al., 2013). Our yields of 33–72 mg/kg fresh biomass are satisfactory for a preliminary proof-of-concept study with no optimization and could be improved by co-expression with protease inhibitors or the viral p19 protein to prevent gene silencing.

In summary, we have shown that the generation of specific antibody fragments against a parasite pathogen can be achieved by panning a phage display library against parasite cells with added bioinformatics vetting. This accessible approach could have potentially broad implications on the selection of other biologically-relevant proteins such as receptors, enzymes, haptens, and vaccine epitopes. We also demonstrate that the chimeric IgG derivatives of the scFv antibody fragment can be expressed in and purified from *N. benthamiana* plants and retain their functionality and specificity against *T. gondii*, making them suitable candidates for the treatment of vulnerable patient groups suffering from toxoplasmosis.

## Supplemental Information

10.7717/peerj.5780/supp-1 Supplemental Information 1 Supplementary Tables.

10.7717/peerj.5780/supp-2 Supplemental Information 2 Supplementary Figures.

10.7717/peerj.5780/supp-3 Supplemental Information 3 Raw blots of Figure 5.

## Additional Information and Declarations

### Data Availability

The following information was supplied regarding data availability: Figshare: https://figshare.com/projects/Plant-derived_chimeric_antibodies_inhibit_the_invasion_of_human_fibroblasts_by_Toxoplasma_gondii/31154.
