# Supplementary material for: Plant-derived chimeric antibodies inhibit the invasion of human fibroblasts by Toxoplasma gondii
Source: PeerJ. 2018 Dec 11;6:e5780. doi: 10.7717/peerj.5780 (PMC6294049; doi:10.7717/peerj.5780)
Supplement: Supplemental Information 1 [file peerj-06-5780-s001.docx]

**Plant-derived chimeric antibodies inhibit the invasion of human fibroblasts by *Toxoplasma gondii***

Sherene Swee Yin Lim^1^, Kek Heng Chua^2^, Greta Nölke^3^, Holger Spiegel^3^, Wai Leong Goh^4^, Sek Chuen Chow^4^, Boon Pin Kee^2^, Rainer Fischer^3^, Stefan Schillberg^3^ & Rofina Yasmin Othman^1,5^

^1^Institute of Biological Sciences, University of Malaya, 50603 Kuala Lumpur, Malaysia.

^2^Department of Biomedical Science, Faculty of Medicine, University of Malaya, 50603 Kuala Lumpur, Malaysia.

^3^Fraunhofer Institute for Molecular Biology and Applied Ecology IME, Forckenbeckstraße 6, 52074 Aachen, Germany.

^4^School of Science, Monash University Malaysia, Jalan Lagoon Selatan, 47500 Bandar Sunway, Selangor Darul Ehsan, Malaysia.

^5^Centre for Research in Biotechnology for Agriculture, University of Malaya 50603, Kuala Lumpur.

Corresponding author:

Rofina Yasmin Othman

Mailing address: Institute of Biological Sciences, University of Malaya, 50603 Kuala Lumpur, Malaysia.

E-mail address: [yasmin@um.edu.my](mailto:yasmin@um.edu.my)

These authors contributed equally to this work.

**Supplementary Table S1: Primer sequences for V_H_ and V_L_ regions amplification.** Primer sequences targeting conserved V regions amplifications, with Sfi1 and Not1 restriction sites underlined and volume of each primer in the primer set mixes indicated.

| **Primer** | Sequence | **Volume (μl)** |
| --- | --- | --- |
| Vκ 5’ / sense (*Sfi* 1) |  |  |
| MSCVK-1 | GGG CCC AGC CGG CCG AGC TCG AYA TCC AGC TGA CTC AGA C | 1 |
| MSCVK-2 | GGG CCC AGC CGG CCG AGC TCG AYA TTG TTC TCW CCC AGT C | 2 |
| MSCVK-3 | GGG CCC AGC CGG CCG AGC TCG AYA TTG TGM TMA CTC AGT C | 5 |
| MSCVK-4 | GGG CCC AGC CGG CCG AGC TCG AYA TTG TGY TRA CAC AGT C | 3.5 |
| MSCVK-5 | GGG CCC AGC CGG CCG AGC TCG AYA TTG TRA TGA CMC AGT C | 4 |
| MSCVK-6 | GGG CCC AGC CGG CCG AGC TCG AYA TTM AGA TRA MCC AGT C | 7 |
| MSCVK-7 | GGG CCC AGC CGG CCG AGC TCG AYA TTC AGA TGA YDC AGT C | 6 |
| MSCVK-8 | GGG CCC AGC CGG CCG AGC TCG AYA TYC AGA TGA CAC AGA C | 1.5 |
| MSCVK-9 | GGG CCC AGC CGG CCG AGC TCG AYA TTG TTC TCA WCC AGT C | 2 |
| MSCVK-10 | GGG CCC AGC CGG CCG AGC TCG AYA TTG WGC TSA CCC AAT C | 3.5 |
| MSCVK-11 | GGG CCC AGC CGG CCG AGC TCG AYA TTS TRA TGA CCC ART C | 8 |
| MSCVK-12 | GGG CCC AGC CGG CCG AGC TCG AYR TTK TGA TGA CCC ARA C | 8 |
| MSCVK-13 | GGG CCC AGC CGG CCG AGC TCG AYA TTG TGA TGA CBC AGK C | 6 |
| MSCVK-14 | GGG CCC AGC CGG CCG AGC TCG AYA TTG TGA TAA CYC AGG A | 2 |
| MSCVK-15 | GGG CCC AGC CGG CCG AGC TCG AYA TTG TGA TGA CCC AGW T | 2 |
| MSCVK-16 | GGG CCC AGC CGG CCG AGC TCG AYA TTG TGA TGA CAC AAC C | 1 |
| MSCVK-17 | GGG CCC AGC CGG CCG AGC TCG AYA TTT TGC TGA CTC AGT C | 1 |
|  |  |  |
| Vκ 3’ / antisense |  |  |
| MSCJK12-B | GGA AGA TCT AGA GGA ACC ACC TTT KAT TTC CAG YTT GGT CCC | 2 |
| MSCJK4-B | GGA AGA TCT AGA GGA ACC ACC TTT TAT TTC CAA CTT TGT CCC | 1 |
| MSCJK5-B | GGA AGA TCT AGA GGA ACC ACC TTT CAG CTC CAG CTT GGT CCC | 1 |
|  |  |  |
| Vλ 5’/ sense (*Sfi* 1) |  |  |
| MSCVL-1 | GGG CCC AGC CGG CCG AGC TCG ATG CTG TTG TGA CTC AGG AAT C | 1 |
|  |  |  |

**Supplementary Table S1: (continuous).**

| Vλ 3’/ antisense-short linker |  |  |
| --- | --- | --- |
| MSCJKL-B | GGA AGA TCT AGA GGA ACC ACC GCC TAG GAC AGT CAG TTT GG | 1 |
|  |  |  |
| V_H_ 5’ / sense |  |  |
| MSCVH1 | GGT GGT TCC TCT AGA TCT TCC CTC GAG GTR MAG CTT CAG GAG TC | 4 |
| MSCVH2 | GGT GGT TCC TCT AGA TCT TCC CTC GAG GTB CAG CTB CAG CAG TC | 4 |
| MSCVH3 | GGT GGT TCC TCT AGA TCT TCC CTC GAG GTG CAG CTG AAG SAS TC | 3 |
| MSCVH4 | GGT GGT TCC TCT AGA TCT TCC CTC GAG GTC CAR CTG CAA CAR TC | 4 |
| MSCVH5 | GGT GGT TCC TCT AGA TCT TCC CTC GAG GTY CAG CTB CAG CAR TC | 7 |
| MSCVH6 | GGT GGT TCC TCT AGA TCT TCC CTC GAG GTY CAR CTG CAG CAG TC | 2 |
| MSCVH7 | GGT GGT TCC TCT AGA TCT TCC CTC GAG GTC CAC GTG AAG CAG TC | 1 |
| MSCVH8 | GGT GGT TCC TCT AGA TCT TCC CTC GAG GTG AAS STG GTG GAA TC | 2 |
| MSCVH9 | GGT GGT TCC TCT AGA TCT TCC CTC GAG GTG AWG YTG GTG GAG TC | 5 |
| MSCVH10 | GGT GGT TCC TCT AGA TCT TCC CTC GAG GTG CAG SKG GTG GAG TC | 2 |
| MSCVH11 | GGT GGT TCC TCT AGA TCT TCC CTC GAG GTG CAM CTG GTG GAG TC | 2 |
| MSCVH12 | GGT GGT TCC TCT AGA TCT TCC CTC GAG GTG AAG CTG ATG GAR TC | 2 |
| MSCVH13 | GGT GGT TCC TCT AGA TCT TCC CTC GAG GTG CAR CTT GTT GAG TC | 1 |
| MSCVH14 | GGT GGT TCC TCT AGA TCT TCC CTC GAG GTR AAG CTT CTC GAG TC | 2 |
| MSCVH15 | GGT GGT TCC TCT AGA TCT TCC CTC GAG GTG AAR STT GAG GAG TC | 2 |
| MSCVH16 | GGT GGT TCC TCT AGA TCT TCC CTC GAG GTT ACT CTR AAA GWG TST G | 5 |
| MSCVH17 | GGT GGT TCC TCT AGA TCT TCC CTC GAG GTC CAA CTV CAG CAR CC | 3.5 |
| MSCVH18 | GGT GGT TCC TCT AGA TCT TCC CTC GAG GTG AAG TTG GAA GTG TC | 0.7 |
| MSCVH19 | GGT GGT TCC TCT AGA TCT TCC CTC GAG GTG AAG GTG ATC GAG TC | 0.7 |
|  |  |  |
| V_H_ 3’ / antisense (*Not*1) |  |  |
| MSCG1ab-B | CCT GCG GCC GCC CAC TAG TGA CAG ATG GGG STG TYG TTT TGG | 3 |
| MSCG3-B | CCT GCG GCC GCC CAC TAG TGA CAG ATG GGG CTG TTG TTG T | 1 |
|  |  |  |
| MSC-F | GCG GGG CCC AGC CGG CCG AGC TCG | 1 |
| RSC-B | GCC TGC GGC CGC ACT AGT GAC AGA | 1 |

**Supplementary Table S2: Anti-*T. gondii* scFv IGHV and IGKV subgroup usage and H / κL-CDR3 motifs.** Candidate scFv clones binding to *T. gondii* were analysed to determine its CDRs and origin of murine germline V-regions using the online V-Quest software provided by the International ImMunoGeneTics data base (IMGT, imgt.cines.fr/textes/vquest/).

| **Clone ID** | **IGHV** | **H chain CDR3** | **H chain CDR3 length (bp)** | **IMGT - H [CDR1.CDR2.CDR3] length** | **GenBank accession no.** | **IGKV** | **L chain CDR3** | **L chain CDR3 length (bp)** | **IMGT - L [CDR1.CDR2.CDR3] length** | **GenBank accession no.** |
| --- | --- | --- | --- | --- | --- | --- | --- | --- | --- | --- |
| **TG64** | 1-4*01 | CAREAWFAYW | 8 | [8.8.8] | AC073561 | 4-70*01 | CHQRSSYPYTF | 9 | [5.3.9] | AJ235943 |
| **TG69** | 14-3*02 | CARLPYW | 5 | [8.8.5] | AJ851868 | 6-23*01 | CQQYSSYHTF | 8 | [6.3.8] | AJ235961 |
| **TG116** | 14-3*02 | CASSHGYEDYFDYW | 12 | [8.8.12] | AJ851868 | 6-13*01 | CQQYSSYPYTF | 9 | [6.3.9] | J00569 |
| **TG130** | 1S29*02 | CARGDGFAYW | 8 | [8.8.8] | J0048 | 6-17*01 | CQQYNSYPYTF | 9 | [6.3.9] | Y15978 |

**Supplementary Table** **S3:** **Results of SAROTUP (TUPScan) (**[**http://immunet.cn/sarotup/**](http://immunet.cn/sarotup/)**) analysis of scFv regions containing target-unrelated peptide (TUP) motif.**

| **ScFv, target-unrelated peptides** | **Matched pattern** | **Brief description** |
| --- | --- | --- |
| TG64 (V_L_ CDR1) MTQSPAILSASPGEKVTMTCRASSSISYMHWYQQKPGTS | S-S-[IL] | Confirmed or suspected binder to immunoglobulin Fc Region. |
| TG64 (V_H_ CDR3) STAYMQLSSLTSEDSAVYYCAREAWFAYWGQGTLVTVSA | S-S-[IL] | Confirmed or suspected binder to immunoglobulin Fc Region. |
| TG69 (V_H_ CDR3) DTSSNTAYLQLSSLTSEDTAVYYCARLPYWGQGTTLTVSS | S-S-[IL] | Confirmed or suspected binder to immunoglobulin Fc Region. |
| TG116 (V_H_ CDR3) YLQLSSLTSEDTAVYYCASSHGYEDYFDYWGQGTTLTVSS | S-S-[IL] | Confirmed or suspected binder to immunoglobulin Fc Region. |

**Supplementary Table S4: Frequency of nucleotides mutated at randomized positions using semi-degenerate primers.** Percentage of each nucleotide residues found at the 4 mutated positions was calculated from a random selection of 20 clones, showing an almost even distribution in nucleotide usage.

|  | **Degenerate code^[[1]](#footnote-1)^** | **Frequency percentage (%)^[[2]](#footnote-2)^** | | | |
| --- | --- | --- | --- | --- | --- |
|  |  | **A** | **C** | **G** | **T** |
| 1^st^ nucleotide | V | 30.0 | 45.0 | 25.0 | - |
| 2^nd^ nucleotide | N | 25.0 | 30.0 | 20.0 | 25.0 |
| 3^rd^ nucleotide | S | - | 45.0 | 55.0 | - |
| 4^th^ nucleotide | N | 25.0 | 30.0 | 20.0 | 25.0 |

V = A or C or G; S = C or G; N = A or C or G or T.

2 Frequency calculated based on number of residues occurring in 20 sequence

1. [↑](#footnote-ref-1)
2. [↑](#footnote-ref-2)
