# Supplementary material for: Plant-derived chimeric antibodies inhibit the invasion of human fibroblasts by Toxoplasma gondii
Source: PeerJ. 2018 Dec 11;6:e5780. doi: 10.7717/peerj.5780 (PMC6294049; doi:10.7717/peerj.5780)
Supplement: Supplemental Information 2 [file peerj-06-5780-s002.docx]

**Plant-derived chimeric antibodies inhibit the invasion of human fibroblasts by *Toxoplasma gondii***

Sherene Swee Yin Lim^1^, Kek Heng Chua^2^, Greta Nölke^3^, Holger Spiegel^3^, Wai Leong Goh^4^, Sek Chuen Chow^4^, Boon Pin Kee^2^, Rainer Fischer^3^, Stefan Schillberg^3^ & Rofina Yasmin Othman^1,5^

^1^Institute of Biological Sciences, University of Malaya, Kuala Lumpur, Malaysia. ^2^Department of Biomedical Science, Faculty of Medicine, University of Malaya, Kuala Lumpur, Malaysia.

^3^Fraunhofer Institute for Molecular Biology and Applied Ecology IME, Aachen, Germany.

^4^School of Science, Monash University Malaysia, Bandar Sunway, Selangor, Malaysia.

^5^Centre for Research in Biotechnology for Agriculture, University of Malaya, Kuala Lumpur, Malaysia.

Corresponding author:

Rofina Yasmin Othman

Mailing address: Institute of Biological Sciences, University of Malaya, 50603 Kuala Lumpur, Malaysia.

E-mail address: [yasmin@um.edu.my](mailto:yasmin@um.edu.my)

These authors contributed equally to this work.


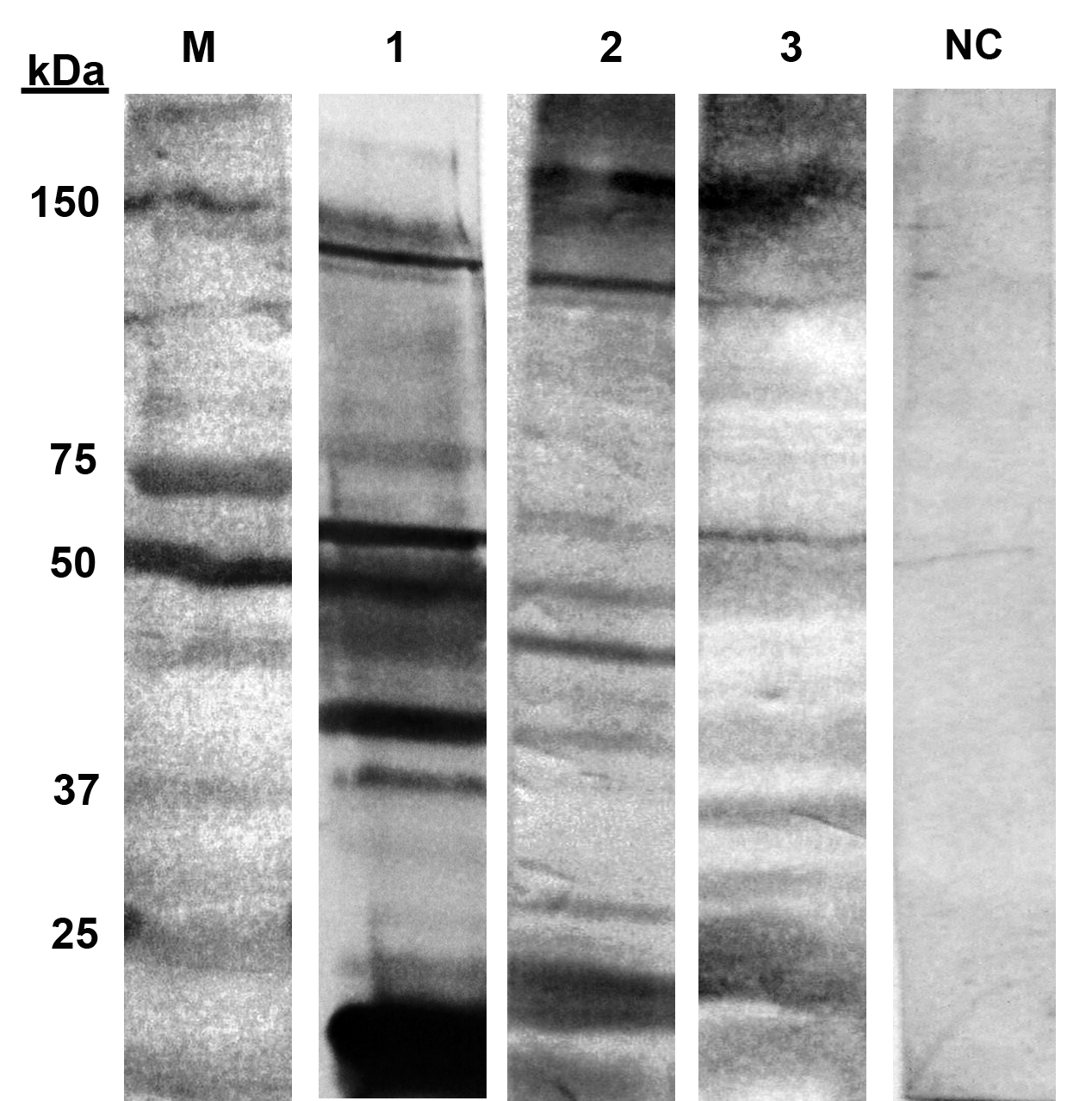


**Supplementary Figure S1: Immunoblot verification of mouse serum immunized against *T. gondii*.** Western blot strips of *T. gondii* parasite separated on SDS-PAGE were probed

with immunized mouse serum individually. The immune mice serum (lanes 1 – 3)

showed IgG reactivity with the parasite antigens. Uninfected mouse serum was used as

negative control (lane NC). The migration of size markers is indicated in kilodaltons

(Prestained protein ladder, Fermentas, SM0671).


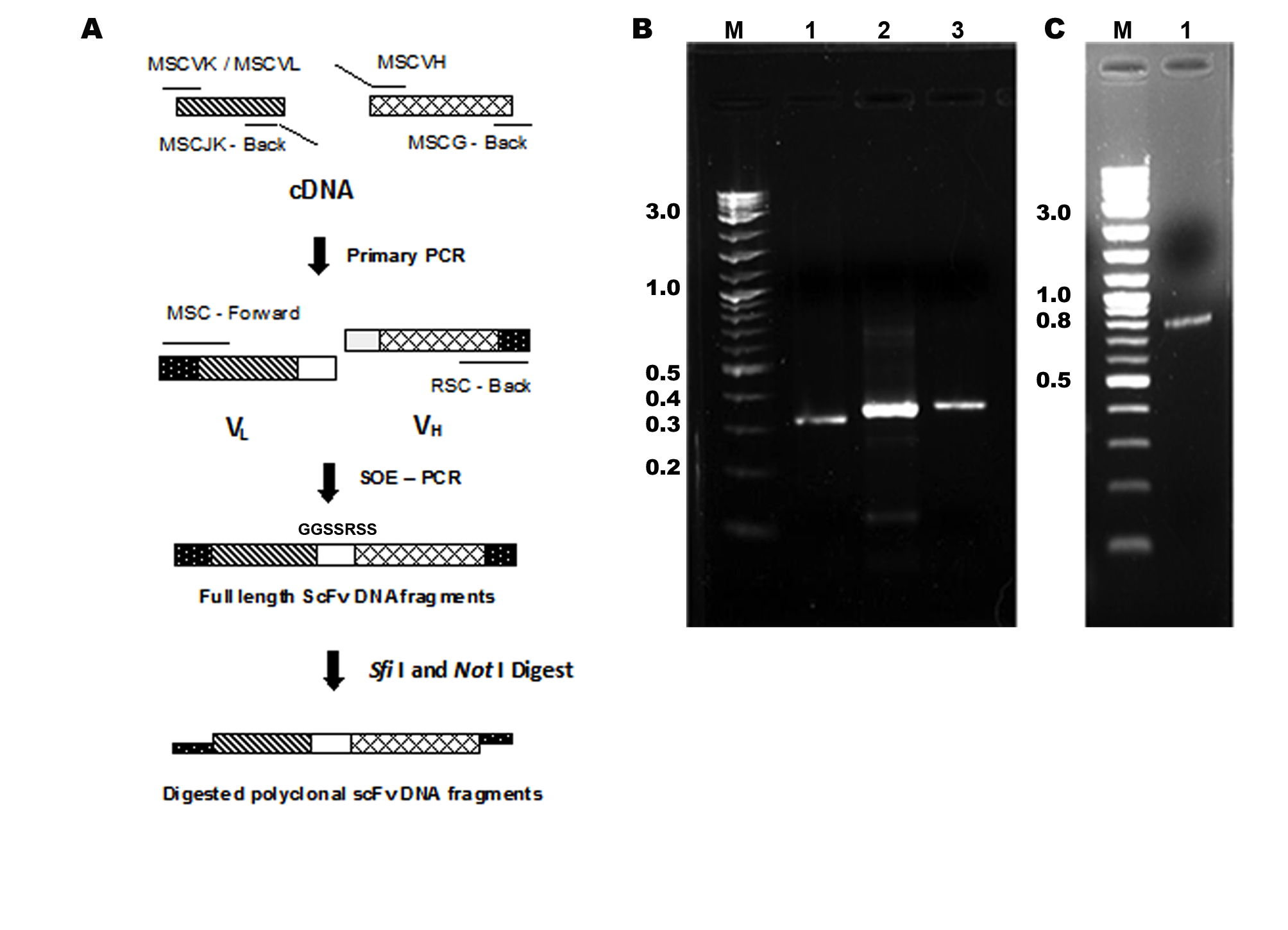


**Supplementary Figure S2: PCR assembly of scFv antibodies. (A)** Strategy of scFv fragment assembly. A short peptide linker (GGSSRSS) was fused between the V_H_ and V_L_ chains and assembled into scFv fragments by splice overlap extension PCR (SOE-PCR). Restriction sites for both *Sfi*I and *Not*I are incorporated at both ends of the scFv for subcloning into the pCANTAB5E vector. **(B and C)** Primary PCR amplification of V-region genes and scFv assembly. B, Amplified V_H_ and V_L_ fragments. Lane 1, V_L_ fragment; lane 2, V_H_ fragment; lane 3, V_H_ size-marker (GE Life Sciences). C, lane 1, Fully-assembled scFv antibody fragment at the expected size of 800 bp. The DNA size marker is indicated on the left in kbp.

**Supplementary Figure S3: Chromatograms of truncated scFv sequences – scFv 18 (Stop**

**codon is indicated in red box).**

**Supplementary Figure S4: Chromatograms of truncated scFv sequences – scFv 48 (Stop**

**codon is indicated in red box).**

**Supplementary Figure S5: Chromatograms of truncated scFv sequences – scFv 103 (Stop codon is indicated in red box).**

**Supplementary Figure S6: Chromatograms of truncated scFv sequences – scFv 109 (Stop codon is indicated in red box).**

**Supplementary Figure S7: Chromatograms of truncated scFv sequences – scFv 118 (Stop codon is indicated in red box).**


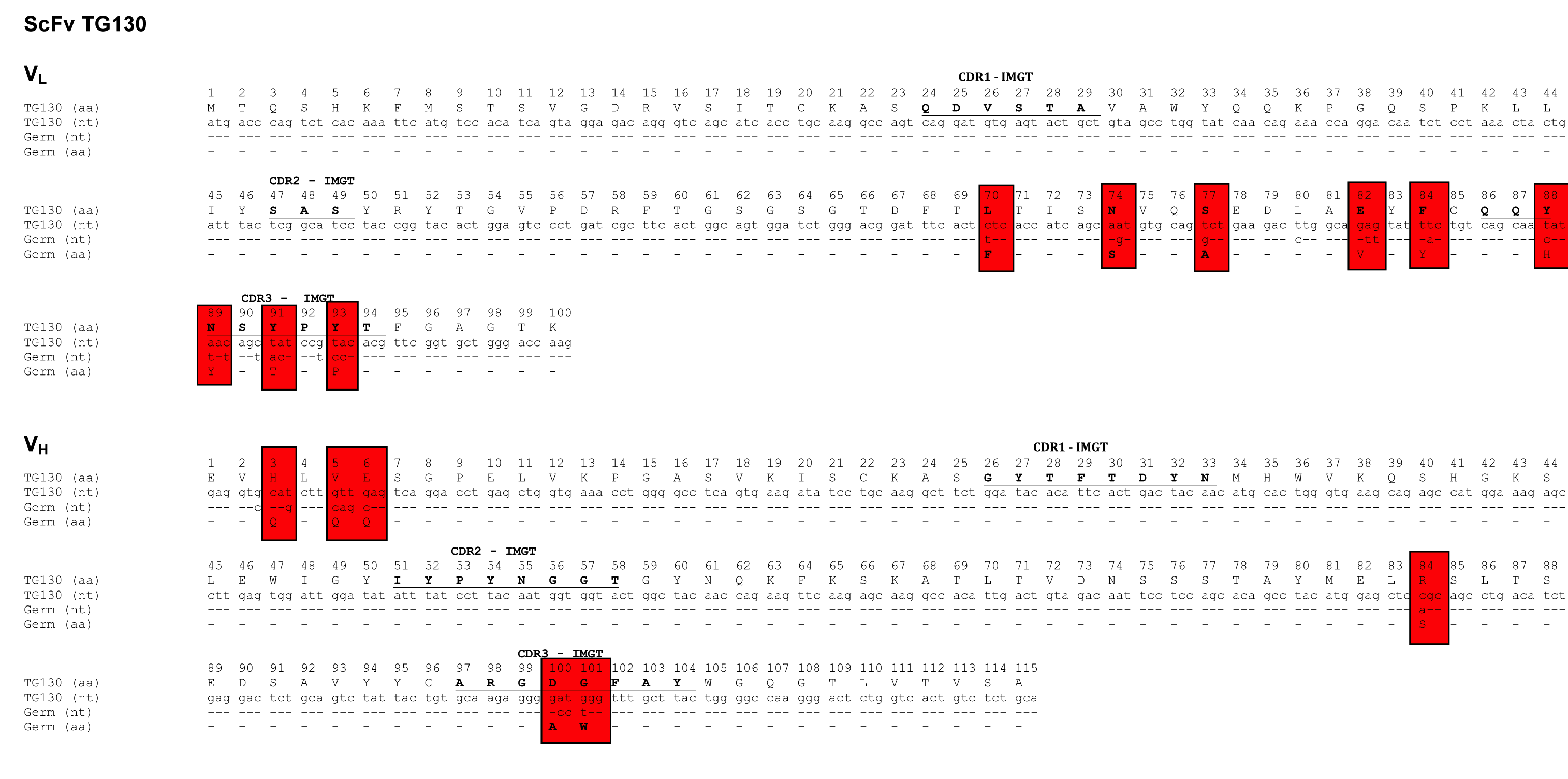


**Supplementary Figure S8: Germline sequence homology alignment with biopanning-selected scFv nucleotide and amino acid sequences.**


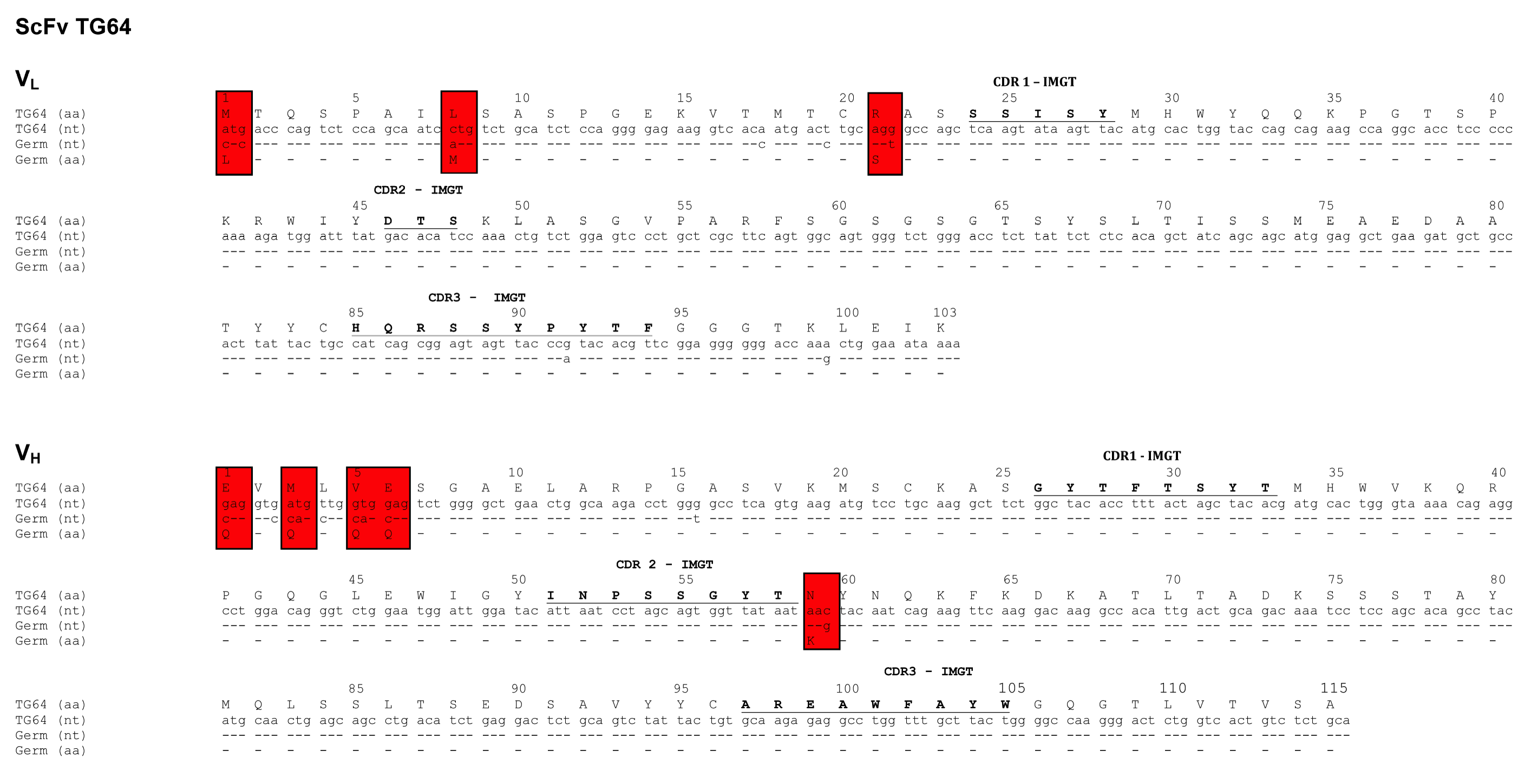


**Supplementary Figure S8: (continuous).**


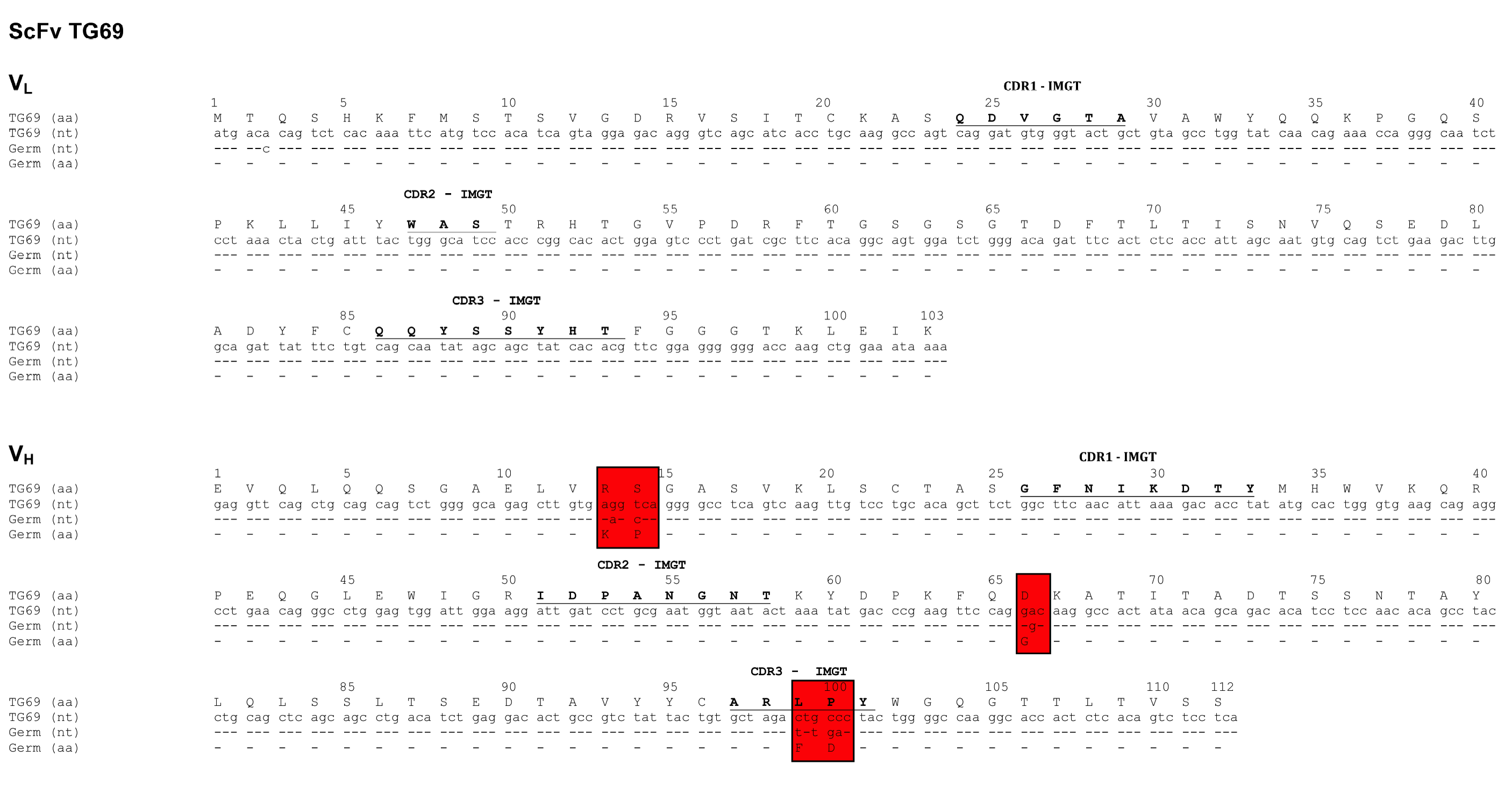


**Supplementary Figure S8: (continuous).**


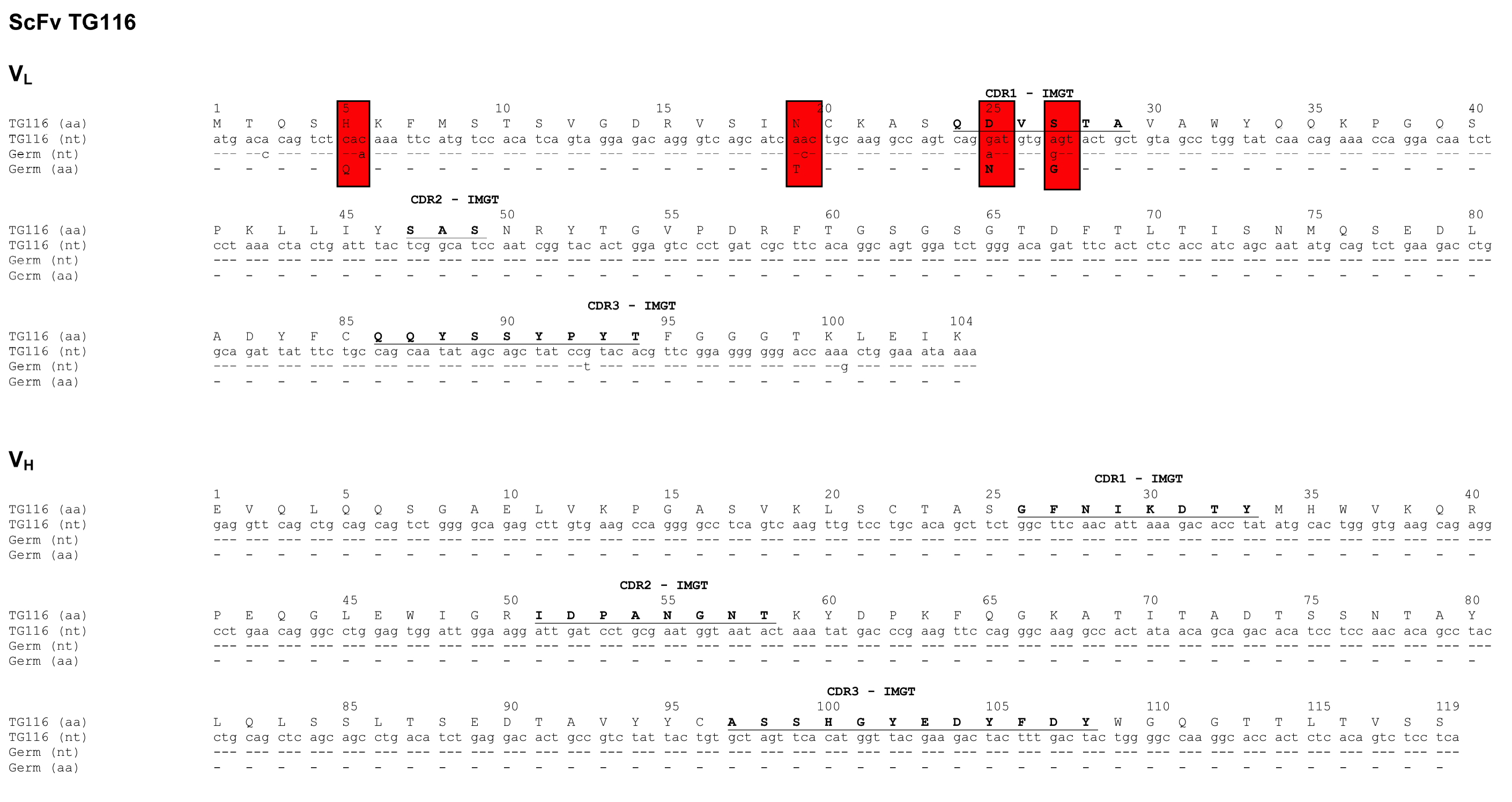


**Supplementary Figure S8: (continuous).**


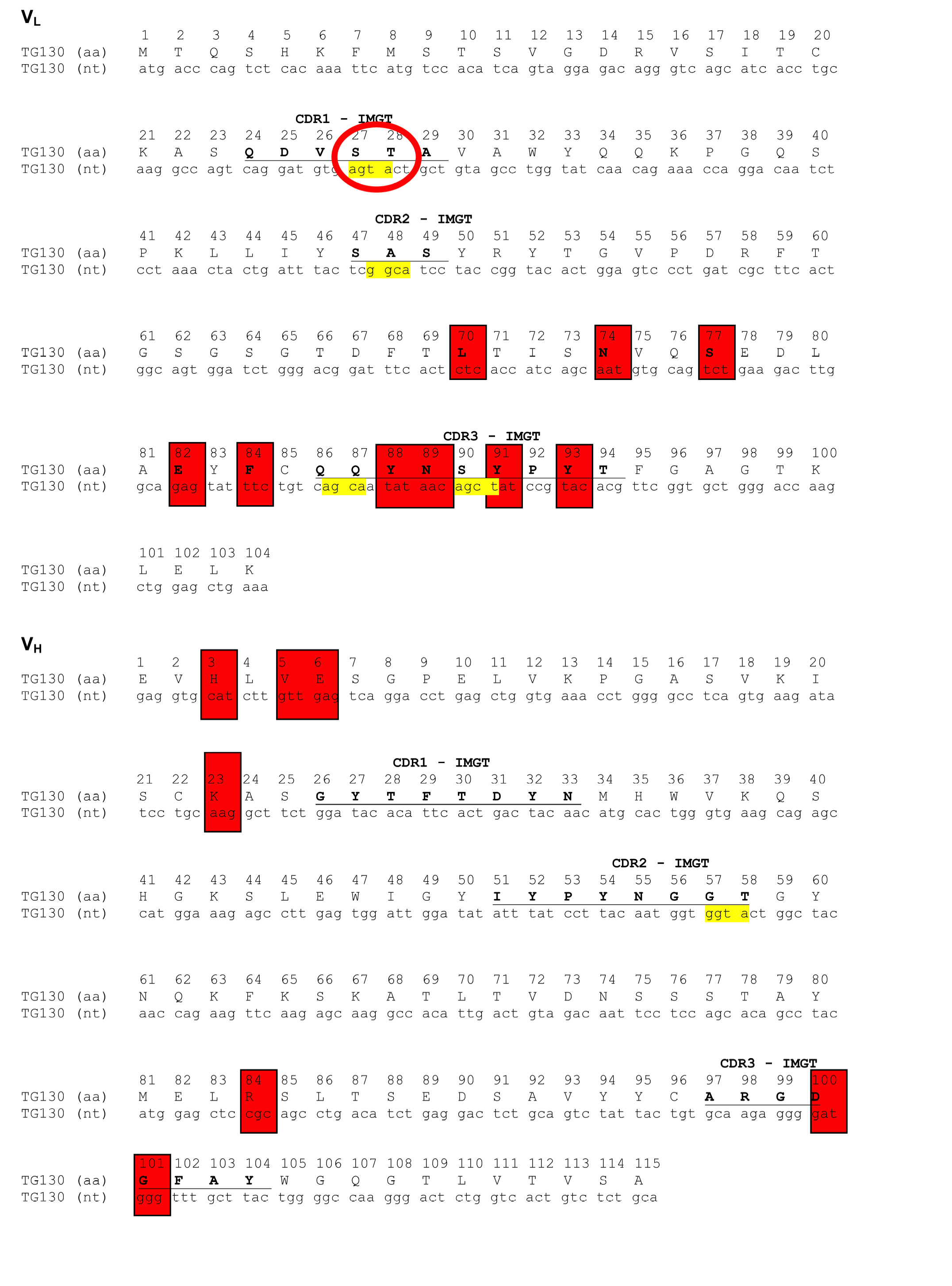


**Supplementary Figure S9: Alignment of nucleotide and amino acid sequences of variable regions of scFv TG130 and targeted affinity mutagenesis.** Regions mutated from germline origin sequences are boxed in red, and RGYW-motif hotspots are highlighted in yellow. The targeted hotspot mutagenesis is circled in red.
