# Supplementary material for: Plant-derived chimeric antibodies inhibit the invasion of human fibroblasts by Toxoplasma gondii
Source: PeerJ. 2018 Dec 11;6:e5780. doi: 10.7717/peerj.5780 (PMC6294049; doi:10.7717/peerj.5780)
Supplement: Supplemental Information 3 [file peerj-06-5780-s003.pdf]

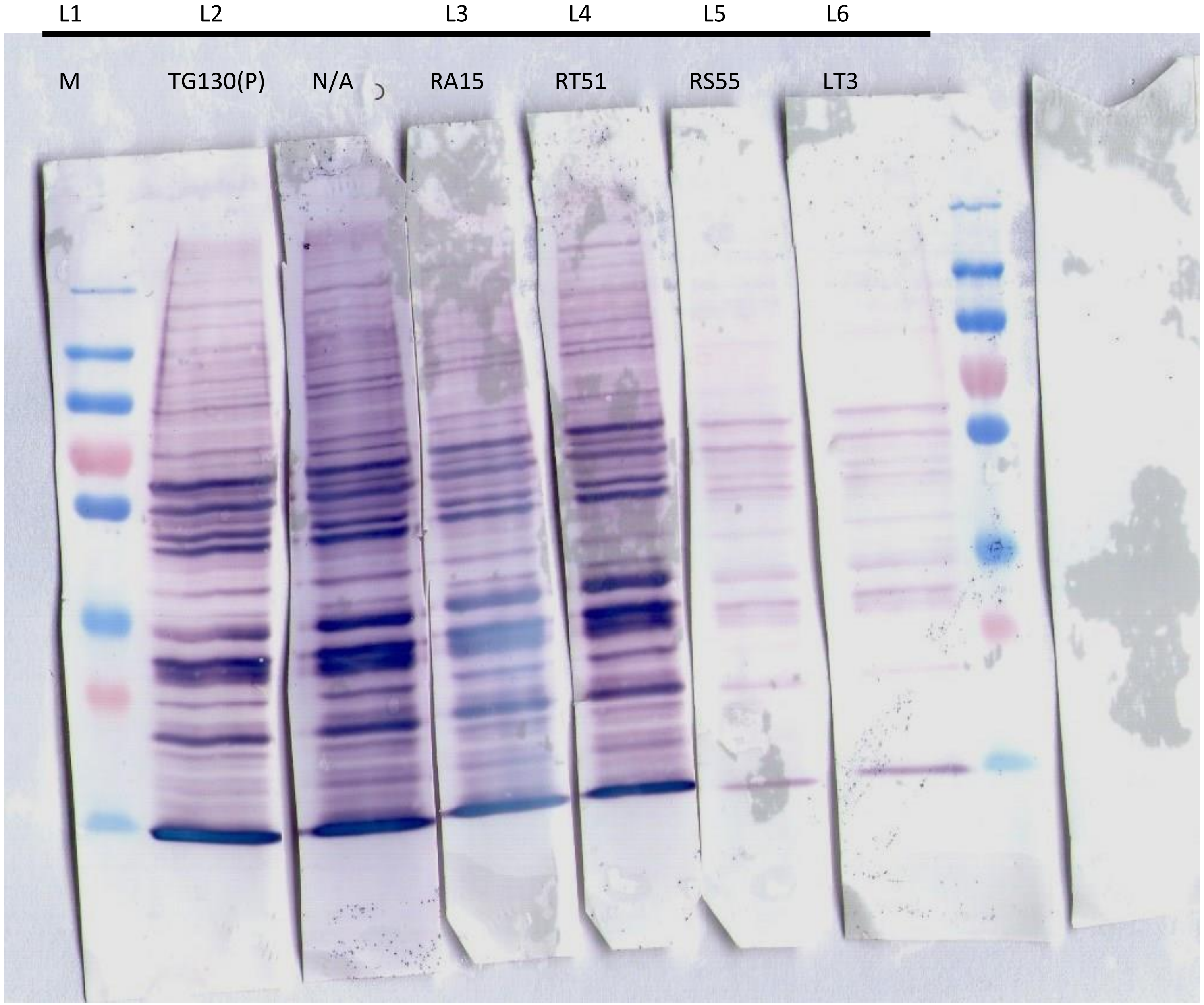

Raw blot of **Figure 5**: Immunoblots of *T. gondii* lysed antigen separated under reducing (R) condition, with the recombinant antibodies used for detection indicated for each blot. L1: marker; L2: TG130(P); L3: RA15; L4: RT51; L5: RS55; L6: LT3.

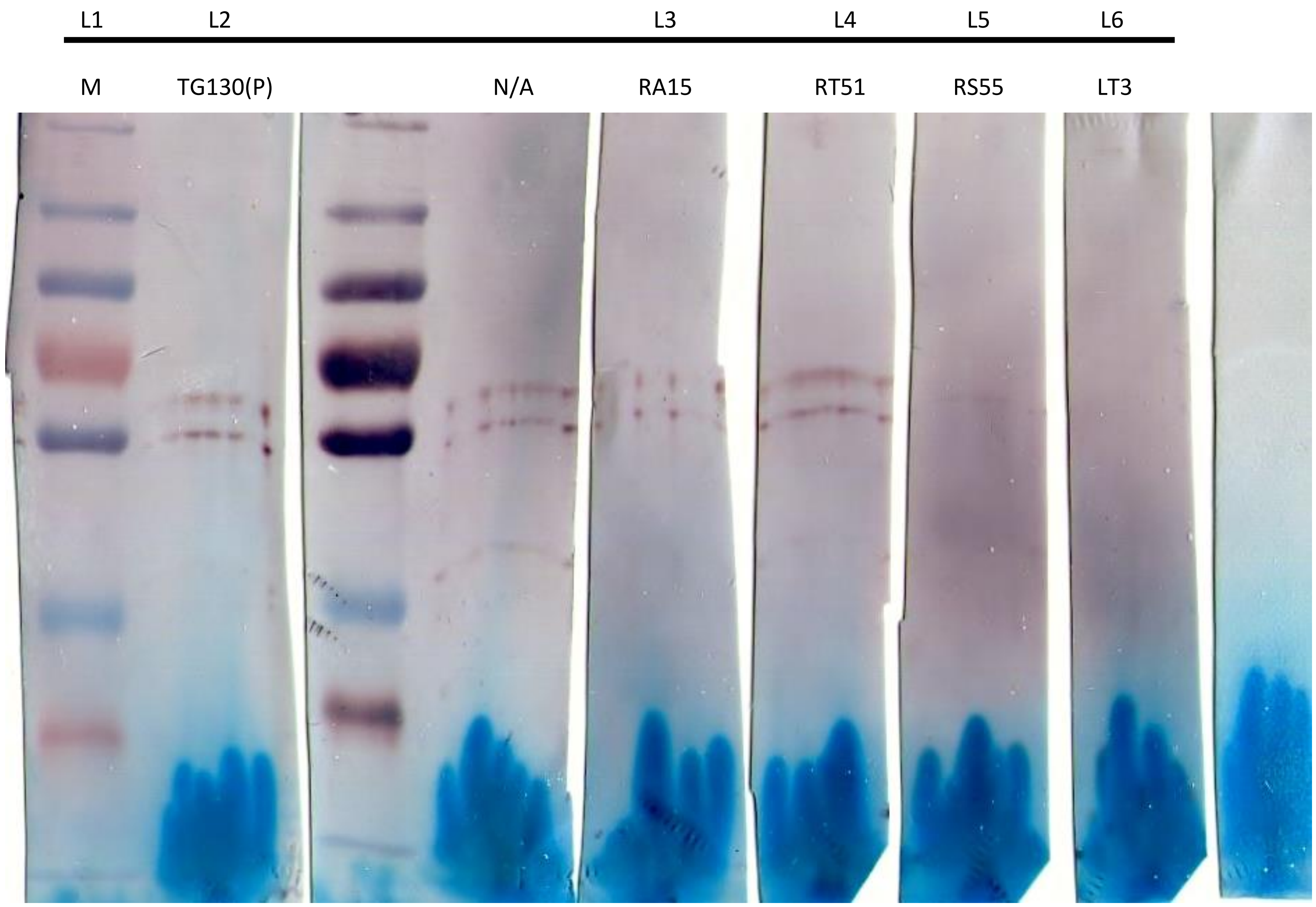

Raw blot of **Figure 5**: Immunoblots of *T. gondii* lysed antigen separated under non-reducing (NR) condition, with the recombinant antibodies used for detection indicated for each blot. L1: marker; L2: TG130(P); L3: RA15; L4: RT51; L5: RS55; L6: LT3.
